# Parvalbumin and Somatostatin Interneurons Contribute to the Generation of Hippocampal Gamma Oscillations

**DOI:** 10.1523/JNEUROSCI.0261-20.2020

**Published:** 2020-09-30

**Authors:** Pantelis Antonoudiou, Yu Lin Tan, Georgina Kontou, A. Louise Upton, Edward O. Mann

**Affiliations:** ^1^Department of Physiology, Anatomy and Genetics, University of Oxford, Oxford, OX1 3PT, United Kingdom; ^2^Oxford Ion Channel Initiative, University of Oxford, Oxford, OX1 3PT, United Kingdom; ^3^Neuroscience, Physiology and Pharmacology, University College London, London, United Kingdom, WC1E 6BT

**Keywords:** gamma, hippocampus, interneuron, oscillation, parvalbumin, somatostatin

## Abstract

γ-frequency oscillations (30-120 Hz) in cortical networks influence neuronal encoding and information transfer, and are disrupted in multiple brain disorders. While synaptic inhibition is important for synchronization across the γ-frequency range, the role of distinct interneuronal subtypes in slow (<60 Hz) and fast γ states remains unclear. Here, we used optogenetics to examine the involvement of parvalbumin-expressing (PV^+^) and somatostatin-expressing (SST^+^) interneurons in γ oscillations in the mouse hippocampal CA3 *ex vivo*, using animals of either sex. Disrupting either PV^+^ or SST^+^ interneuron activity, via either photoinhibition or photoexcitation, led to a decrease in the power of cholinergically induced slow γ oscillations. Furthermore, photoexcitation of SST^+^ interneurons induced fast γ oscillations, which depended on both synaptic excitation and inhibition. Our findings support a critical role for both PV^+^ and SST^+^ interneurons in slow hippocampal γ oscillations, and further suggest that intense activation of SST^+^ interneurons can enable the CA3 circuit to generate fast γ oscillations.

**SIGNIFICANCE STATEMENT** The generation of hippocampal γ oscillations depends on synchronized inhibition provided by GABAergic interneurons. Parvalbumin-expressing (PV^+^) interneurons are thought to play the key role in coordinating the spike timing of excitatory pyramidal neurons, but the role distinct inhibitory circuits in network synchronization remains unresolved. Here, we show, for the first time, that causal disruption of either PV^+^ or somatostatin-expressing (SST^+^) interneuron activity impairs the generation of slow γ oscillations in the ventral hippocampus *ex vivo*. We further show that SST^+^ interneuron activation along with general network excitation is sufficient to generate high-frequency γ oscillations in the same preparation. These results affirm a crucial role for both PV^+^ and SST^+^ interneurons in hippocampal γ oscillation generation.

## Introduction

γ oscillations (30-120 Hz) are a common feature of active cortical networks, which have been proposed to contribute to local gain control (Cardin et al., 2009; Sohal et al., 2009; Sohal, 2016) and facilitate transmission between synchronized neuronal assemblies (Fries, 2005; Akam and Kullmann, 2010; Fries, 2015). While the function of γ oscillations remains debated (Burns et al., 2011; Butler and Paulsen, 2014; Bastos et al., 2015; Ray and Maunsell, 2015; Womelsdorf and Everling, 2015; Lasztóczi and Klausberger, 2016; Sohal, 2016), changes in these rhythms continue to act as a useful marker of function and dysfunction in cortical circuit operations (Bragin et al., 1995; Fries et al., 2001; Herrmann and Demiralp, 2005; Uhlhaas and Singer, 2006, 2010; Basar-Eroglu et al., 2007; Yamamoto et al., 2014; Spellman et al., 2015). There is a general consensus that the generation of γ rhythms depends on the spiking of inhibitory interneurons, which synchronize the firing of excitatory pyramidal cells via fast synaptic inhibition (Whittington et al., 1995; Penttonen et al., 1998; Csicsvari et al., 2003; Hájos et al., 2004; Hasenstaub et al., 2005; Mann et al., 2005b; Bartos et al., 2007; Buzsáki and Wang, 2012; Kim et al., 2016; Chen et al., 2017; Veit et al., 2017). Specifically, parvalbumin-expressing (PV^+^) interneurons, which target the perisomatic domain of pyramidal neurons, are thought to play the key role in generating and maintaining γ oscillations in the brain (Penttonen et al., 1998; Csicsvari et al., 2003; Hájos et al., 2004; Gloveli et al., 2005; Mann et al., 2005b; Hájos and Paulsen, 2009; Tukker et al., 2013; Cardin, 2016). PV^+^ interneurons are adapted for fast synchronization of network activity, as they resonate at γ frequencies and exert strong perisomatic inhibition that is capable of precisely controlling spike timing (Pike et al., 2000; Pouille and Scanziani, 2001; Cardin et al., 2009; Bartos and Elgueta, 2012; Hu et al., 2014; Kohus et al., 2016).

Recently, a selective role for PV^+^ interneurons in γ-frequency synchronization has been challenged by several studies performed in the primary visual cortex (Chen et al., 2017; Veit et al., 2017; Hakim et al., 2018). In this brain region, it was shown that dendrite-targeting somatostatin-expressing (SST^+^) interneurons were the main contributors for the generation of slow γ oscillations, while PV^+^ interneurons were more important for higher frequency synchronization (Chen et al., 2017). Previous studies have found analogous roles for SST^+^ and PV^+^ interneurons in low- and high-frequency network synchronization (Beierlein et al., 2000; Gloveli et al., 2005; Tukker et al., 2007; Craig and McBain, 2015). However, it is not yet clear whether SST^+^ interneurons might play a more generic role in the generation of slow γ oscillations across brain areas.

The hippocampus displays both slow and fast γ rhythms during theta activity, with slow γ generated in CA3 and fast γ propagated from entorhinal cortex (Bragin et al., 1995; Colgin et al., 2009; Schomburg et al., 2014; Lasztóczi and Klausberger, 2016). The circuitry for slow γ oscillations is preserved in hippocampal slices (Fisahn et al., 1998), and these models have been used extensively to show that PV^+^ interneurons are strongly phase-coupled to γ oscillations, and contribute to rhythmogenesis (Hájos et al., 2004; Gloveli et al., 2005; Mann et al., 2005b; Gulyás et al., 2010). However, the majority of interneurons are phase-coupled to ongoing slow γ oscillations (Hájos et al., 2004; Gloveli et al., 2005; Oren et al., 2006), and it may be that SST^+^ interneurons play an important role in synchronizing PV^+^ networks. Indeed, whether specific classes of CA3 interneuron are necessary and sufficient for the generation of slow γ oscillations has not yet been tested. Here, we took advantage of optogenetic techniques (Nagel et al., 2003; Boyden et al., 2005; Chow et al., 2010) to test the involvement of PV^+^ and SST^+^ interneurons in cholinergically induced γ oscillations in the CA3 of acute hippocampal slices.

## Materials and Methods

### 

#### 

##### Transgenic mice

All procedures were performed according to the United Kingdom Animals Scientific Procedures Act 1986 and the University of Oxford guidelines. Adult (older than 8 weeks, both male and female) PV-cre (B6;129P2-Pvalbtm1(cre)Arbr/J), PV-cre-Ai9 (PV-Cre x Gt ROSA (CAG-tdTomato) Hze/J), and SST-cre mice (Sst tm2.1(cre)Zjh/J) were used for all experiments.

##### Stereotaxic viral injections

Anesthesia was induced in mice with 4% isoflurane/medical oxygen mixture (2 L per min). The area around the head was shaved and cleaned in preparation for scalp incision. Anesthesia was subsequently maintained using 1.5%-2.5% isoflurane at a rate of 2 L per min. Before the onset of the procedure, a cocktail of systemic perioperative analgesics (Metacam [meloxicam] 1 mg/kg and Vetergesic [buprenorphine] 0.1 mg/kg) and a local analgesic (Marcaine [bupivacaine] 10 mg/kg) were administered subcutaneously (Oxford University Veterinary Services). Following, antibiotic solution was applied on the head, and an incision of the scalp was performed that allowed a small craniotomy to be made. A 33/34-gauge needle was attached on a Hamilton Microliter Syringe and used to inject the virus solution at a rate of ≈100 nl/min (viral concentration ≈ 10^12^ genome copies per ml). After every injection, the needle was left stationary for at least 3 min to allow diffusion of virus in the surrounding area. The virus solution was injected with the aid of a stereotaxic frame into ventral CA3 area of hippocampus (2.7 mm caudal and 2.75 mm lateral from bregma). A total of 600-800 nl was injected at two depths (300-400 nl at 3.1 mm and 300-400 nl at 2.7 mm). Following the injection, local analgesic (Marcaine 10 mg/kg) was applied on the incised scalp before it was sutured. The animals were then transferred in a heating chamber and allowed to recover. The animals were monitored, and welfare scored in the following days to ensure that they properly recovered after surgery. Injected mice were assessed for viral expression after a minimum of 3 weeks. All viral constructs were acquired from Vector Core Facilities, Gene Therapy Center (North Carolina, UNC). Viral constructs used are as follows: AAV5-EF1a-DIO-ChR2(H134R)-mCherry, AAV5-EF1a-DIO-ChR2(H134R)-eYFP, AAV-EF1a-DIO-Arch3.0-EYFP, AAV-Ef1a-DIO-hChR2(E123T-T159C)-p2A-mCherry-WPRE (Karl Deisseroth), and AAV-CAG-FLEX-ArchT-GFP (Ed Boyden).

##### *Ex vivo* brain slice preparation

Mice were anesthetized using 4% isoflurane (Oxford University Veterinary Services) and were killed by decapitation after the pedal reflex was abolished. Brains were extracted in warm (30°C–35°C) sucrose solution (34.5 mm NaCl, 3 mm KCl, 7.4 mm MgSO_4_·7H_2_O, 150 mm sucrose, 1 mm CaCl_2_, 1.25 mm NaH_2_PO_4_, 25 mm NaHCO_3_, and 15 mm glucose), and transverse hippocampal slices of 350 μm thickness were cut using a Leica vibratome (VT 1200S) (Huang and Uusisaari, 2013). Slices were then immediately placed in an interface storing chamber containing warm (30°C–35°C) aCSF (126 mm NaCl, 3.5 mm KCl, 2 mm MgSO_4_-7H_2_O, 1.25 mm NaH_2_PO_4_, 24 mm NaHCO_3_, 2 mm CaCl_2_, and 10 mm glucose) at least 1 h to equilibrate. All solutions were bubbled with 95% O_2_ and 5% CO_2_ beginning 30 min before the procedure until the end of the experiment.

##### Electrophysiology

Extracellular recordings were conducted in an interface recording chamber at 33°C–34°C. Visualization of the slices and electrode placement were performed using a Wild Heerbrugg dissection microscope. Local field potentials (LFPs) were recorded by inserting a borosilicate glass electrode filled with aCSF (tip resistance = 1-5 mΩ) in CA3 pyramidal layer. Data were acquired and amplified (10×) by Axoclamp 2A (Molecular Devices). The signal was further amplified 100× and low pass filtered at 1 kHz (LPBF-48DG, NPI Electronic). The signal was then digitized at 5 kHz by a data acquisition board (ITC-16, InstruTECH) and recorded from the IgorPro (Wavemetrics). γ oscillations were induced by the application of 5 μm carbachol (Cch). The LFP signal was quantified using real-time FFT analysis, and oscillations were detected by a peak in the power spectrum at low-band frequencies (25-49 Hz). For unit recordings, a linear 16-channel tungsten multielectrode array (MEA; MicroProbes) was lowered in the CA3 subfield. The array channels had 100 μm spacing to ensure full coverage of the hippocampus. The MEA was mounted on an RHD2132 Amplifier board and connected to the RHD2000 USB Interface Board (Intan Technologies). Data were acquired at a rate of 20 kHz using the RHD2000 rhythm software (Intan Technologies).

Intracellular recordings were always conducted in a single submerged chamber (26°C–32°C) using borosilicate glass pipettes (5–12 MOhm). The signal was acquired through the MultiClamp 700B amplifier (Molecular Devices) and digitized at a rate of 10 kHz by a data acquisition board (ITC-18, InstruTECH) and was then recorded using the Igor Pro 6.37 software. The signals were low pass filtered (Bessel) at 10 kHz for current-clamp mode and 3 kHz for voltage clamp mode. Slice and cell visualization was achieved using oblique illumination and monitored through a Hamatatsu Orca-ER digital camera. Filtered white LED (460 ± 30 nm, 1.53 mW, Thor Labs) via epi-illumination was used to activate channelrhodopsin (ChR2). Filtered white LED (525 ± 20 nm, 1.45 mW, Thor Labs) via epi-illumination was used to activate archaerhodopsin (Arch). For a power of 1.5 mW, the light intensity in the illuminated area was 3.68 mW/mm^2^. Cell-attached recordings were performed in current-clamp mode (Multiclamp software) using glass pipettes filled with aCSF. For whole-cell current-clamp recordings, pipettes were filled with internal solution containing 110 mm K gluconate, 40 mm HEPES, 2 mm ATP-Mg, 0.3 mm GTP-NaCl, and 4 mm NaCl (3-4 mg/ml biocytin, Sigma Millipore). For whole-cell voltage-clamp recordings, pipettes were filled with internal solution containing 140 mm cesium methanesulfonate, 5 mm NaCl, 10 mm HEPES, 0.2 mm EGTA, 2 mm ATP-Mg, 3 mm GTP-Na, and 5 mm QX-314 (3-4 mg/ml biocytin). Series resistance compensation was not performed in all cells included for analysis. For perforated patch recordings, the tip of the pipette was filled with a KCl-containing solution (150 mm KCl and 10 mm HEPES, pH 7.2-7.3; osmolality 300 mOsmol/kg). The rest of the pipette was filled with the same KCl solution containing 5 μm gramicidin D (1:1000 DMSO dilution, Sigma Millipore) and 10 μm fluorescein (Sigma Millipore) to visualize whether there was spontaneous rupture of the membrane during patching experiments.

##### Light delivery

For photoexcitation (ChR2) experiments, light illumination was delivered through a fiber optic using a blue LED (470 ± 20 nm, Thorlabs, M470F3; max power at fiber optic tip = 10 mW). For photoinhibition (Arch) experiments, light illumination was delivered through a fiber optic by a green LED (530 ± 30 nm, Thorlabs, M530F2; maximum power at fiber optic tip = 4.25 mW) and with an amber LED (595 ± 20 nm, Doric, maximum power at fiber optic tip = 5 mW). LED module output was controlled using the Igor Pro 6.37 software. Laser photoinhibition experiments were also performed with a green laser (MatchBox series, 532 ± 0.5 nm, maximum power at fiber optic that was used ∼40 mW). In these experiments, the data were acquired at a rate of 10 kHz using Igor Pro 6.37. The laser was operated manually, and the light duration was recorded using an Arduino Uno board that created a digital time stamp. Experiments were only included if the laser illumination duration was between 19.6 and 20.7 s. The area of light illumination was estimated to have a diameter of 1-2 mm; therefore, for a power of 10 mW, the light intensity was between 0.8 and 3.2 mW/mm^2^.

##### Histology and imaging

After electrophysiological recordings, acute brain slices were fixed in 4% PFA overnight. Slices were kept in PBS (1.37 mm NaCl, 2.7 mm KCl, 10 mm Na_2_HPO_4_, 2 mm KH_2_PO_4_) at 4°C for short-term storage. For biocytin labeling, the slices were washed with 1× PBS 3-4 times and permeabilized with freshly prepared 0.3%-Triton 1× PBS for 4-5 h. Streptavidin conjugated to AlexaFluor-488 (Invitrogen, S32355) in PBS-Triton 0.3% (1:500) was incubated overnight at 4°C. The slices were then washed 4-5 times in PBS for 2 h. Slices were mounted on glass slides using mounting media (Dako). Confocal images (1024 × 1024) were acquired on an LSM700 upright confocal microscope (Carl Zeiss) using the 10× air objective and digitally captured using the default LSM acquisition software. Pyramidal cell reconstruction was performed on neuron studio and simple neurite tracer plugin on Fiji. Fluorescence expression was quantified using 40 pixel-wide line profiles through the layers of CA3 in Fiji, with background subtraction, and the signal normalized to the background.

##### Analysis of LFPs

In order to characterize and analyze the oscillations, a Hanning window was applied and the power spectra were calculated as the normalized magnitude square of the FFT (Igor Pro 6.37). The 50 and 100 Hz frequencies were not included in the analysis to exclude the mains noise and its harmonic component. The oscillation amplitude was quantified (1) by measuring the peak of the power spectrum termed as peak power and (2) by measuring the area below the power spectrum plot in the γ-band range (20-100 Hz) termed as power area. The peak frequency of the oscillation was obtained by measuring the frequency at which the peak of the power spectrum occurred in the γ-band range. In order to quantify when Cch-induced oscillations were abolished on light stimulation, and to exclude the peak frequencies of those oscillations from further analysis, one of the two criteria had to be met. First, an autocorrelation of the oscillations was computed and was fitted with a Gabor function $\left(f(x)=(A*cos(2\pi *f*x))*e(-x^{2}/2*tau)\right)$. The first criterion was met if the resulting Gabor fit had a linear correlation coefficient, *r* > 0.7 and $\sqrt{f*tau}$ > 0.1 (>0.15 for frequencies higher than 50 Hz). The second criterion was a power area larger than 125 μV^2^ in the range of ± 5 Hz of the peak frequency. The power area was always included in the analysis, even if oscillations were abolished. The power spectrum analysis for *de novo* oscillations was performed in the range of 52-149 Hz with the only criterion for oscillation presence being that the power area in ± 5 Hz of the peak frequency was larger than 40 μV^2^. Hilbert transforms were used to obtain instantaneous γ magnitude for sinusoidal modulation of γ oscillations (bandpass filtered 20-120 Hz). For visualization purposes, the magnitude of the continuous wavelet transform was used normalized by max value (Morlet wavelet; ω = 6).

##### Spike detection and analysis

Unit detection was performed using custom-written procedures in MATLAB (2015-17, The MathWorks). Extracellular spikes from the 16-channel MEA were detected as described previously by Quiroga and colleagues (Quian Quiroga et al., 2004; Quian Quiroga, 2009). Briefly, the MEA data were processed with an elliptical bandpass filter (for spike detection: fourth order, 300-3000 Hz, for spike sorting: second order, 300-6000 Hz). Spikes were detected as signals exceeding 5 SDs of the noise $5*\sigma_{n}=median\{|x|/0.6745\}$. Signals that exceeded 10 times the SD of the detected spike amplitudes were eliminated as artifacts/population spikes. Subsequently, spikes that had peaks occurring at the same time (<0.1 ms) across channels were grouped together as one unit. This prevented detection of the same unit more than once. Clustering of the detected spikes was performed using custom-written procedures in Igor Pro 6.37. A spike sorting procedure adapted from Fee et al. (1996) was used to explore whether neurons displaying specific spike waveforms were selectively recruited by optogenetic stimulation. Briefly, spike metrics were converted into *z* scores, over-clustered using an in-built *k*-means algorithm, and progressively aggregated if the intercluster distance was <2.5 and merging did not produce more violations of refractory period of 2 ms. Analysis was performed on the clustered spikes, with autocorrelation and cross-correlation plots used to validate the clustering procedure. Spike metrics from the average waveform for each cluster were used to identify different waveform types via a *k*-means algorithm. This clustering procedure is likely to be conservative, and underestimate the firing rate of individual neurons, but was deemed sufficiently robust to detect any bias in optogenetic recruitment. A single-unit cluster was identified if it (1) had <1.4% of its total spike waveforms within 2 ms of its refractory period and (2) consisted of >800 members. When a cluster did not obey these criteria, it was merged with other clusters that had similar action potential waveforms giving rise to a multiunit cluster.

Clusters were identified as expressing ChR2 if the spike rate in the first 100 ms of the step stimulus was 3 SDs above the baseline spike rate. The remaining clusters were classified based on the delay between the negative and positive peaks in the average waveform as fast spiking (<0.6 ms) or regular spiking (≥0.6 ms). The Activation Index was calculated over the last second of the step stimulus as the difference between the light-induced and baseline spikes rates divided by their sum, and designed to measure sustained firing. The Theta Modulation Index was calculated as the rank correlation coefficient between the spike time histogram and the theta-modulated amplitude of the light stimulus.

##### Computational modeling

The activity in populations of excitatory and PV^+^ inhibitory neurons was modeled using Wilson-Cowan firing rate equations (Devalle et al., 2017) as follows:

(1) $$\tau_{m}\dot{R}=-\dot{R}+G\left(D_{syn\_total}+D_{ext}\right)$$

where $\tau_{m}$ is the membrane time constant, *R* is the mean firing rate (and the overdot indicates a derivative taken with respect to time), $D_{syn\_total}$ is the total synaptic drive, $D_{ext}$ is the external drive to mimic Cch induced depolarization or optogenetic manipulation, and $G\left(x\right)$ is the population response function. See the following:

(2) $$G\left(x\right)=\frac{1}{\sqrt{2}\pi \tau_{m}}.\sqrt{x+\sqrt{x^{2}+\Delta^{2}}}$$

This includes the parameter Δ to represent heterogeneity, which was set at 0.3 throughout.

The activity in a population of SST^+^ interneurons was modeled using macroscopic equations derived from quadratic integrate-and-fire neurons (Devalle et al., 2017) as follows:

(3) $$\tau_{m}\dot{R}=\frac{\Delta }{\pi \tau_{m}}+2RV$$

(4) $$\tau_{m}\dot{V}=V^{2}-{\left(\pi \tau_{m}R\right)}^{2}+D_{syn\_total}+D_{ext}$$

which include the influence of mean membrane voltage (V), and enable oscillations in a lumped model of inhibitory neurons. $\tau_{m}$ was 10 ms for excitatory cells, and 5 ms for both populations of inhibitory cell.

For all three neuronal populations, the total synaptic drive was the sum of excitatory and inhibitory inputs each cell population received, with each modeled as exponentially decaying synapses as follows:

(5) $$D_{syn\_total}=\sum_{i}D_{{syn}_{i}}$$

(6) $$D_{{syn}_{i}}=w_{i}\tau_{m}S_{i}$$

(7) $$\tau_{s{yn}_{i}}{\dot{S}}_{i}=-S_{i}+R_{{pre}_{i}}$$

where w is the synaptic weight (Table 1), S is the variable for synaptic activation, $\tau_{syn}$ is the synaptic time constant (Table 1), and $R_{pre}$ is the respective presynaptic spike rate. The relative synaptic weights were based on synaptic currents recorded in excitatory and inhibitory neurons during cholinergically induced γ oscillations (Oren et al., 2006). The synaptic time constants were tuned to give ∼35 Hz oscillations across a range of $I_{ext}$ in excitatory cells (6-12).

**Table 1.** Synaptic parameters

Presynaptic	Synaptic weights (postsynaptic)			Synaptic time constants (ms) (postsynaptic)		
	E	PV	SST	E	PV	SST
E	10	30	10	10	3	5
PV	−15	−10	---	7	7	---
SST	−15	---	−10	10	---	10

The differential equations were solved in MATLAB 2019a using the ode23tb function and a 0.1 ms time step. The signal used to analyze rhythmic activity was the inhibition in the *E* cells, calculated as the sum of wS for the input from PV^+^ and SST^+^ neurons, as cholinergically induced LFP oscillations in the hippocampus *ex vivo* appear to reflect inhibitory currents in pyramidal neurons (Mann et al., 2005a; Oren et al., 2010). Similar to the analysis of the LFP, the frequency was only reported if: (1) the Gabor fit to the autocorrelation showed r > 0.7 and $\sqrt{f*tau}$ > 0.1, and (2) the peak power in the γ range was >1. The peak power was calculated for all simulations.

##### Statistics

Repeated-measures ANOVA was performed in SPSS 24 with a Greenhouse-Geisser correction where required (i.e., significance in Mauchly's test for sphericity) and followed by Bonferroni-corrected *post hoc* paired *t* tests. Linear correlations, circular correlations, and Bonferroni-corrected one-sample *t* tests were performed using Igor Pro 6.37. Spearman's Rank correlations were performed in SPSS 24. Scatter-bar charts were generated using PRISM 7. Circular statistics of spike phase relative to ongoing oscillations in the LFP were calculated using in-built functions in Igor Pro 6.37. The measurements of spiking rates deviated from normality and were analyzed using nonparametric statistical tests performed in SPSS 24: differences between cell types were analyzed using Kruskal–Wallis test, followed by *post hoc* Dunn's test, with Bonferroni correction for multiple comparisons. Differences across stimulus types (step and theta) were analyzed using the Wilcoxon signed rank test, and the significance of modulation indices analyzed using the one-sample Wilcoxon signed rank test (H_0_ = 0). Error bars in graphs indicate the SEM, unless explicitly stated otherwise.

## Results

### PV^+^ interneuron activity is necessary for cholinergically induced γ oscillations in hippocampal CA3

**Figure 1.**
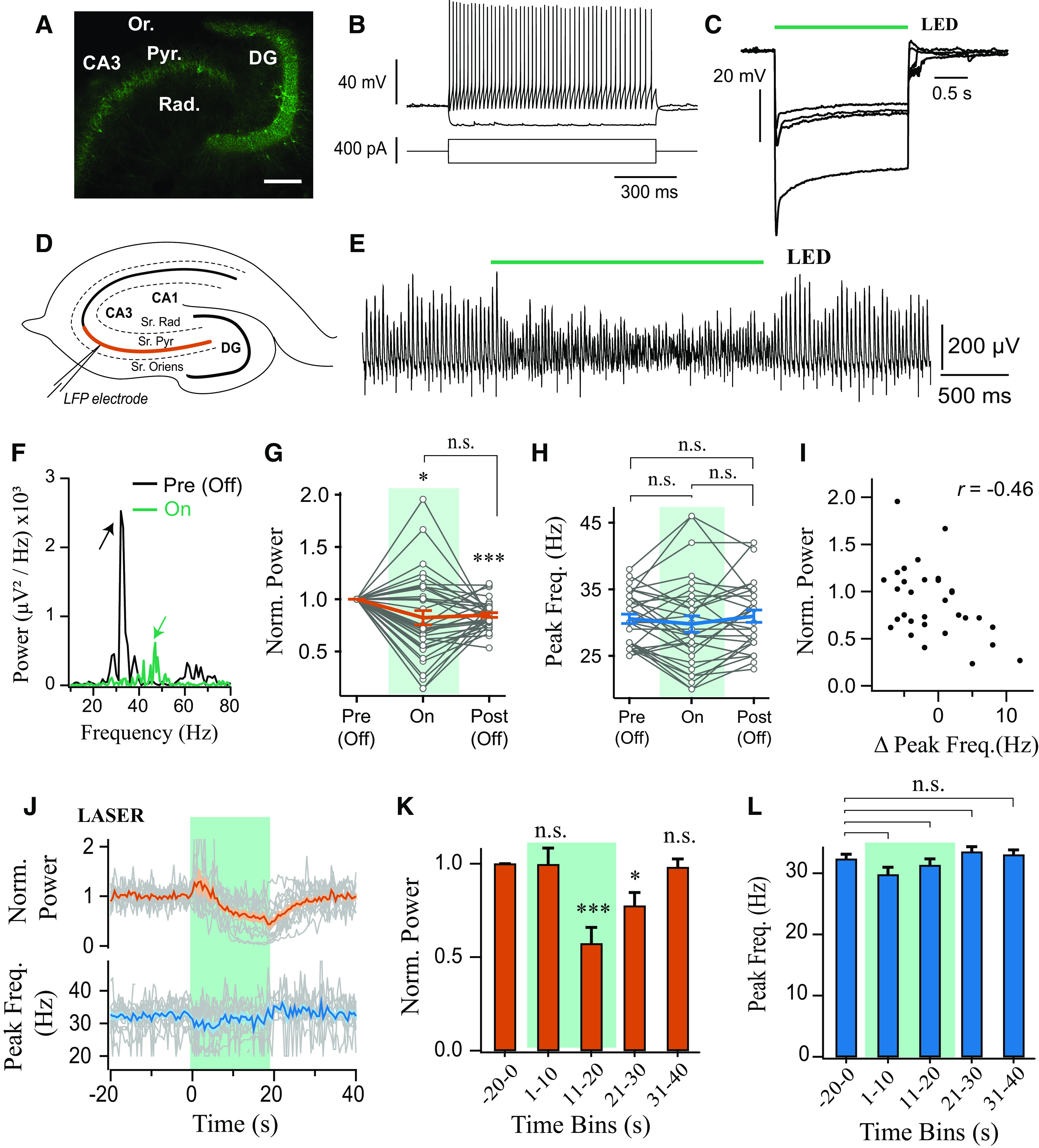
Sustained photoinhibition of PV^+^ interneurons suppresses the power of γ oscillations. ***A***, Confocal image of ventral hippocampus slice from a PV-cre mouse injected intrahippocampally with AAV-Arch3 eYFP. CA3, Cornu ammonis 3; DG, dentate gyrus; Pyr., stratum pyramidale; Rad., stratum radiatum; Or., stratum oriens. Scale bar, 200 μm. ***B***, Current-clamp recording of an ArchT-GFP-expressing PV^+^ cell from CA3 area, showing responses to depolarizing and hyperpolarizing current steps, and fast-spiking phenotype. ***C***, Potent hyperpolarization of four PV^+^ interneurons during green light illumination in aCSF (1.45 mW). ***D***, Illustration of the electrophysiological setup, with colored line indicating the region of CA3 stratum pyramidale from which recordings were obtained. ***E***, Cholinergically induced oscillations (5 μm Cch) were suppressed during PV^+^ interneuron photoinhibition (LED, 530 nm, ∼4.25 mW). ***F***, Representative power spectra before (black) and during (green) LED illumination (arrows indicate peaks in the power spectra). ***G***, Power area in the 20-100 Hz band normalized to baseline (Pre (Off)) during (On) and after LED stimulation (Off (Post)) (*n* = 35). ***H***, Peak frequency for experiments when the oscillation was not abolished (*n* = 31 of 35). ***I***, Change in power area plotted against change in peak frequency. ***J***, Stronger photoinhibition was achieved using high power laser illumination (∼18.6 mW). Top, Change in power area normalized to baseline. Bottom, Peak frequency of the oscillation calculated in 1 s bins across experiments (*n* = 14). ***K***, Mean change in power area normalized to baseline (*n* = 14). ***L***, Mean peak frequency for trials when the oscillation was not abolished (*n* = 13). *p < 0.05, **p < 0.01, ***p < 0.001. n.s., not significant, *p* ≥ 0.05. Changes in peak frequency were analyzed using repeated-measures ANOVA, followed by *post hoc* paired *t* tests with correction for multiple comparisons. Solid brackets represent paired *t* tests. Asterisks above symbols or bars represent one-sample t-test versus normalised baseline. Gray lines indicate single experiments.

**Figure 2.**
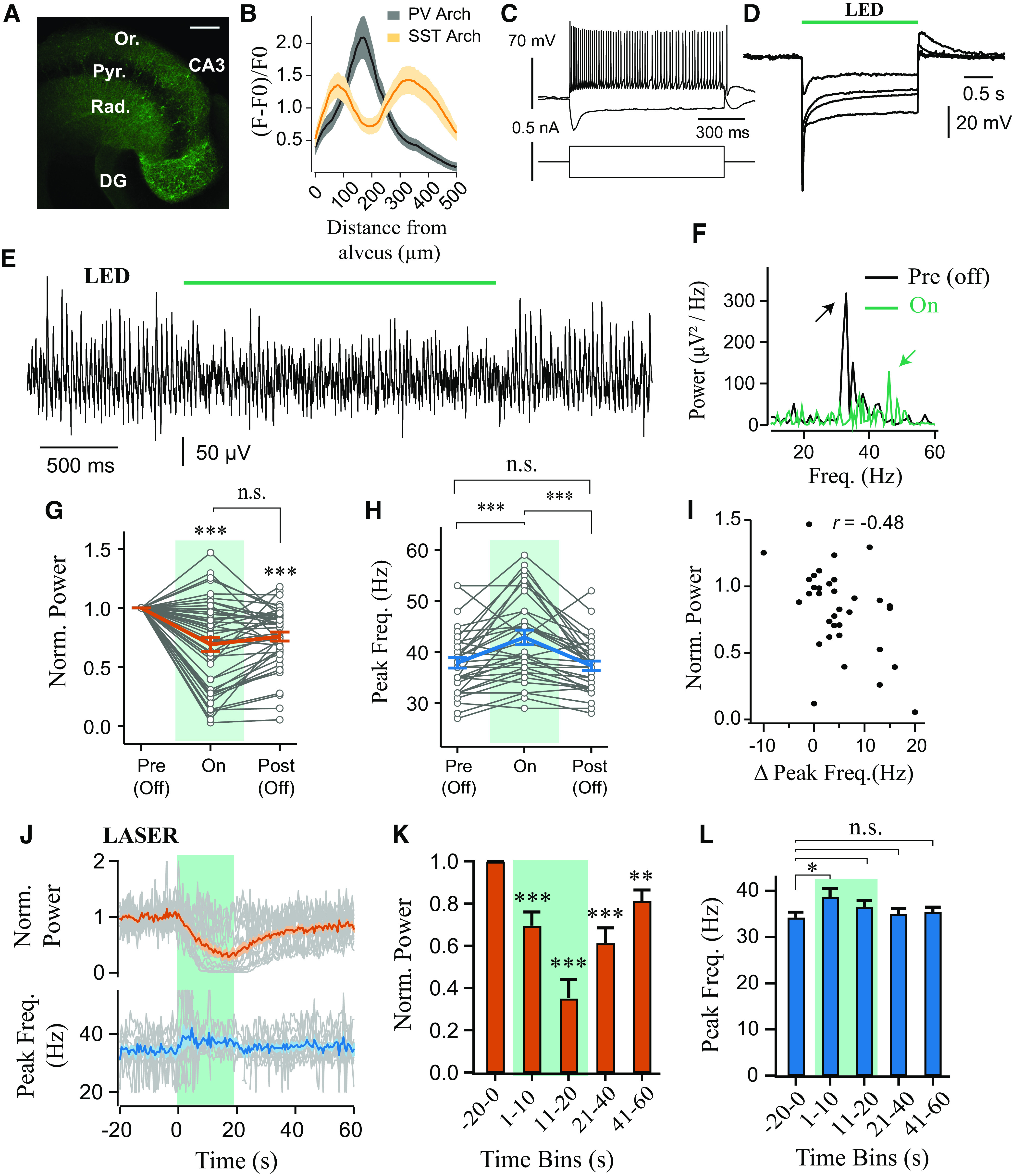
Sustained photoinhibition of SST^+^ interneurons suppresses γ power and increases frequency. ***A***, Confocal image of ventral hippocampus slice from SST-cre mice with eYFP-Arch3 expression. Scale bar, 200 μm. ***B***, Quantification of fluorescence expression profile in PV (*n* = 18) and SST (*n* = 21) ArchT-GFP-expressing slices. ***C***, Current-clamp recording of an SST^+^ cell from CA3 area, showing responses to depolarizing and hyperpolarizing current steps. ***D***, Potent hyperpolarization of four SST^+^ interneurons during green light illumination (1.45 mW). ***E***, Representative LFP recordings illustrating effect of SST^+^ interneuron photoinhibition (LED, 530 nm, ∼4.25 mW). ***F***, Representative power spectra before (black) and during (green) LED illumination (arrows indicate peaks in the power spectra). ***G***, Power area in the 20-100 Hz band normalized to baseline (Pre (Off)) during (On) and after LED stimulation (Post (Off)) (*n* = 44). ***H***, Peak frequency for experiments when the oscillation was not abolished (*n* = 33 of 44). ***I***, Change in power area plotted against change in peak frequency. ***J***, Effects of sustained laser illumination (∼18.6 mW) on normalized power area (top) and peak frequency (bottom) calculated in 1 s bins (*n* = 17). ***K***, Mean normalized power area (*n* = 17). ***L***, Mean peak frequency for trials when the oscillation was not abolished (*n* = 11 of 17). *p < 0.05, **p < 0.01, ***p < 0.001. n.s., not significant, *p* ≥ 0.05. Solid brackets represent paired *t* tests. Asterisks above symbols or bars represent one-sample *t* test versus normalised baseline. Gray lines indicate single experiments.

In order to test whether the activity of PV^+^ interneurons is necessary for the generation of slow hippocampal γ oscillations, we took advantage of optogenetic photoinhibition (Chow et al., 2010). We injected PV-cre mice with AAV carrying the inhibitory proton pump archaerhodopsin (Arch3-eYFP or ArchT-GFP). Expression of Arch in PV-cre mice was restricted to the pyramidal cell layer indicating preferential expression in perisomatic targeting PV^+^ interneurons (Figs. 1*A*, 2*B*) (Somogyi and Klausberger, 2005; Royer et al., 2012; Hu, Gan and Jonas, 2014). Intracellular recordings performed in opsin-expressing cells demonstrated that these cells were fast-spiking and that sustained light illumination was able to produce robust hyperpolarization, indicating functional expression of Arch in PV^+^ interneurons (Fig. 1*B*,*C*).

γ oscillations were induced in hippocampal slices from PV-Arch mice in area CA3 using bath application of the cholinergic agonist Cch (5 μm). LFP recordings from the CA3 pyramidal cell layer revealed robust γ oscillations that were centered at ∼30-40 Hz (Fig. 1*D–F*), as has been reported previously (Fisahn et al., 1998; Hájos et al., 2004; Mann et al., 2005b). In each optogenetic experiment, the photoexcitation protocols were repeated multiple times (4-10 trials) and the mean response reported. Overall, sustained photoinhibition of PV^+^ interneurons using LED illumination (<5 mW) significantly decreased γ power area (0.82 ± 0.068 of baseline period, *t* = 2.59, *p* = 0.029, one-sample *t* test; Fig. 1*E*,*G*), although increases in power were observed in some slices (Fig. 1*G*). A significant suppression was also observed in the period of 0.5-1.5 s following light illumination termination (0.85 ± 0.022 of baseline period, *t* = 6.70, *p* < 0.001, one-sample *t* test; Fig. 1*G*). The light-induced changes in γ power were reversible, as there were no significant changes in the γ power area recorded during the baseline periods across trials (*F*_(4,116)_ = 0.68, *p* = 0.61, repeated-measures ANOVA). In addition, the changes in γ power were not accompanied by a consistent alteration in γ frequency (*F*_(1.44,43.23)_ = 1.25, *p* = 0.288, repeated-measures ANOVA; Fig. 1*H*), although there was a significant correlation between the changes in frequency and power area (*t* = 2.77, *p* = 0.01, Pearson correlation, Fig. 1*I*), suggesting a consistent modulation of endogenous oscillatory activity.

While LED photoinhibition of PV^+^ interneurons significantly modulated γ power, the oscillations did not collapse. Pyramidal neurons make strong recurrent connections with PV^+^ interneurons (Mann et al., 2005a; Oren et al., 2006; Hofer et al., 2011; Packer and Yuste, 2011; Bartos and Elgueta, 2012; Kohus et al., 2016), and it might be hard to break these feedback loops with photoinduced inhibitory currents. To test this possibility, we used long-lasting laser illumination with the prospect of biochemically silencing PV^+^ interneurons, by preventing synaptic release via terminal alkalization (El-Gaby et al., 2016). PV^+^ interneurons expressing ArchT-GFP were illuminated with sustained green laser light (532 nm, ∼18 mW for 20 s). Similar to the LED experiments, there were inconsistent network responses to PV^+^ interneuron photoinhibition at the beginning of laser illumination (1.00 ± 0.087 of baseline period, *t* = 0.04, *p* = 1.00, one-sample *t* test; Fig. 1*J*,*K*). However, the power of the oscillation consistently decreased during sustained laser illumination (0.57 ± 0.086 of baseline period, *t* = 5.00, *p* < 0.001, one-sample *t* test; Fig. 1*J*,*K*) and remained suppressed in first 10 s following laser stimulation (Post1: 0.78 ± 0.071 of baseline period, *t* = 3.17, *p* = 0.030, one-sample *t* test; Fig. 1*J*,*K*), but eventually recovered (Post2: 0.98 ± 0.04 of baseline period, *t* = 0.43, *p* = 1.00, one-sample *t* test; Fig. 1*J*,*K*). There was no consistent effect on the frequency of the oscillations (*F*_(1.93,23.21)_ = 7.29, *p* = 0.004, repeated-measures ANOVA; paired *t* tests to baseline, *t* < 2.692, *p* > 0.078, Fig. 1*J*,*L*). Laser illumination of PV^+^ interneurons expressing only control fluorophore did not alter γ oscillation power nor frequency (data not shown). This slow and selective process of decreasing γ power is consistent with biochemical silencing of synaptic terminals (El-Gaby et al., 2016). These results further support the importance of PV^+^ interneuron activity in generating γ oscillations in hippocampal area CA3 (Hájos et al., 2004; Mann et al., 2005b; Gulyás et al., 2010; Tukker et al., 2013). Residual γ oscillations following photoinhibition of PV^+^ interneurons may reflect incomplete transfection of the PV^+^ network or the presence of a distinct oscillatory circuit.

### SST^+^ interneurons are necessary for Cch-induced γ oscillations in hippocampal area CA3

To examine whether SST^+^ interneuron activity is also required during Cch-induced γ oscillations in CA3, we injected the AAV-Arch vector (Arch3-eYFP or ArchT-GFP) intrahippocampally in SST-cre mice. Expression of Arch was restricted to the strata oriens, radiatum, and lacunosum moleculare (Fig. 2*A*,*B*), suggesting expression in SST^+^ dendrite-targeting interneurons (Ma et al., 2006; Lovett-Barron et al., 2012; Muller and Remy, 2014; Urban-Ciecko and Barth, 2016). Whole-cell recordings were performed in opsin-positive cells and indicated functional expression of Arch (*n* = 4; Fig. 2*C*,*D*). SST^+^ interneurons often showed a pronounced sag during current- and light-induced hyperpolarization (Fig. 2*C*,*D*), and could show rebound spikes following light pulses and steps (data not shown), but remained hyperpolarized throughout photostimulation.

Unlike the experiments with PV^+^ photoinhibition, sustained photoinhibition of SST^+^ interneurons using LED illumination (<5 mW) reliably decreased γ oscillation power (0.69 ± 0.057 of baseline period, *t* = 5.40, *p* < 0.001, one-sample *t* test; Fig. 2*E–G*), which remained suppressed in the immediate period following SST^+^ interneuron photoinhibition (0.76 ± 0.039 of baseline period, *t* = −6.26745, *p* < 0.001, one-sample *t* test; Fig. 2*G*). This post-light suppression was reversed from trial to trial (*F*_(4,164)_ = 2046, *p* = 0.048, repeated-measures ANOVA; all paired *t* tests, *t* > 2.81, *p* > 0.07). In addition, light stimulation significantly modulated oscillation frequency (*F*_(1.25,39.97)_ = 22.60, *p* < 0.001, repeated-measures ANOVA), with an increase in frequency from 37.79 ± 1.083 Hz to 43.00 ± 1.466 Hz during light stimulation (*t* = 4.74, *p* < 0.001, paired *t* test), which reversed following light offset (Fig. 2*H*). There was also a significant correlation between the changes in frequency and power area of the oscillations (*t* = 3.11, *p* = 0.004, Pearson correlation; Fig. 2*I*), suggesting again a consistent modulation of endogenous oscillatory activity.

In the first 10 s of stronger laser illumination (532 nm, ∼18 mW, 20 s total duration), similar effects were observed as in the LED experiment. Specifically, the power of Cch γ oscillations decreased (0.70 ± 0.064 of baseline, *t* = 4.76, *p* < 0.001, one-sample *t* test), and the peak frequency increased (34.22 ± 1.191 Hz to 38.60 ± 1.868 Hz, *t* = 3.93, *p* = 0.011, paired *t* test; repeated-measures ANOVA, *F*_(1.79,17.87)_ = 4.61, *p* = 0.028; Fig. 2*J–L*). During the second half of the stimulation period (10-20 s), γ power was strongly suppressed (0.35 ± 0.090 of baseline, *t* = 7.23, *p* < 0.001, one-sample *t* test; Fig. 2*J–L*), often resulting in oscillation collapse (7 of 13 slices). This could indicate that silencing SST^+^ interneurons is sufficient to disrupt the hippocampal network during γ oscillations and that SST^+^ interneuron activity is necessary for proper maintenance of Cch-induced oscillations in the CA3 area of the hippocampus. Moreover, the robust upregulation of γ oscillation peak frequency (Fig. 2*J*,*L*) suggests that SST^+^ interneurons can exert strong control over the frequency of slow γ oscillations.

### Rhythmic synchronization of the hippocampal network by perisomatic and dendritic inhibition

**Figure 3.**
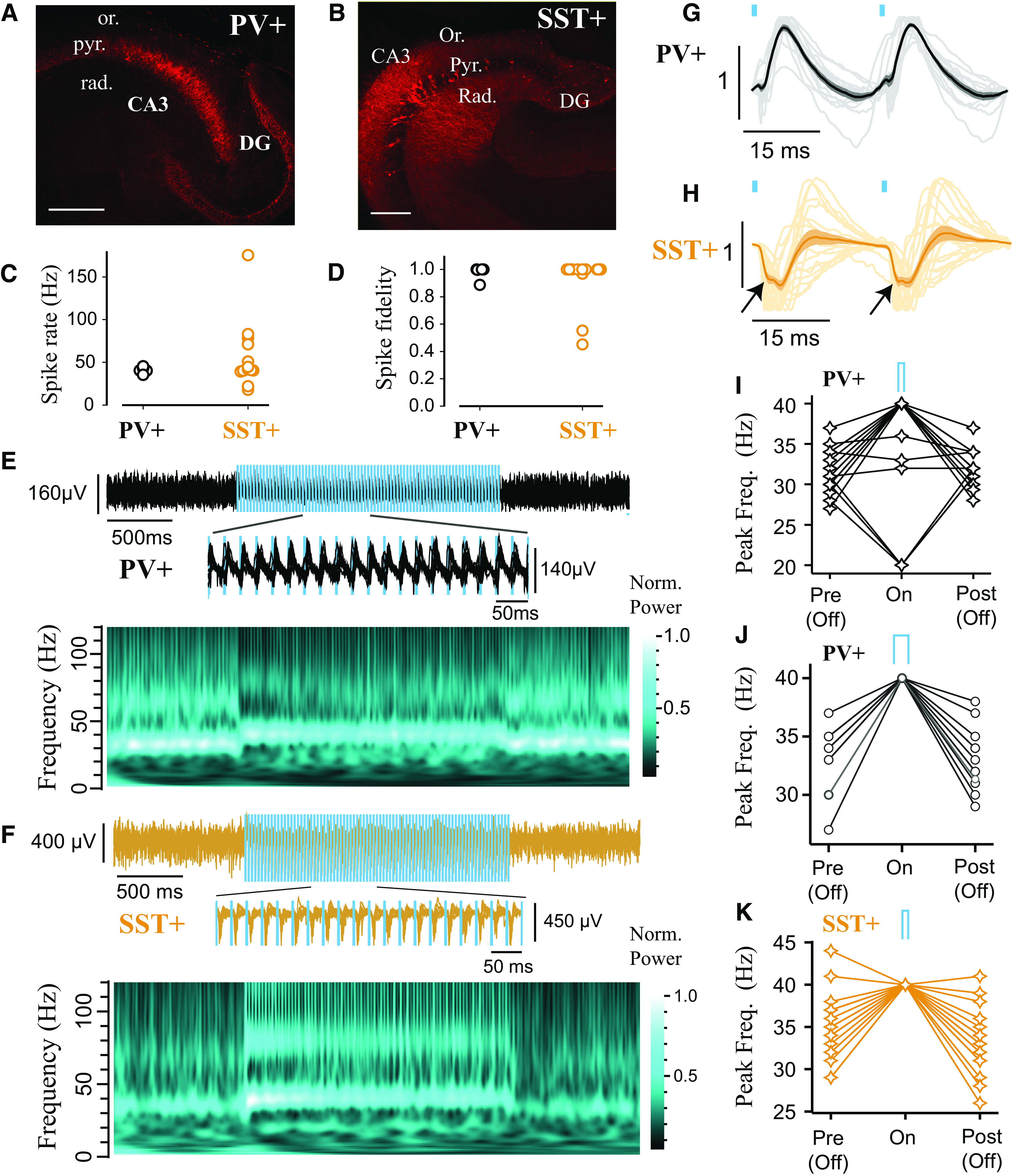
Rhythmic photoexcitation of either PV^+^ or SST^+^ interneurons entrains Cch-induced γ oscillations. ***A***, ***B***, Confocal images of ventral hippocampus (350 μm slice) from PV-cre (***A***) and SST-cre (***B***) mice with mCherry-ChR2 expression. Scale bar, 200 μm. ***C***, ***D***, Spiking responses of PV^+^ and SST^+^ interneurons during 40 Hz light pulses (1-5 ms pulse width), characterized in terms of spike rate (***C***) and spike fidelity (***D***). ***E***, ***F***, Entrainment of Cch-induced oscillations to 40 Hz light pulses in PV-cre (***E***) and SST-cre (***F***) mice expressing mCherry-ChR2 (1 ms pulse width; blue light illumination at 5.5 mW), shown in LFP traces (top) and normalized wavelet spectrum (bottom). Brighter colors represent larger magnitudes. ***G***, ***H***, Normalized average waveform following two consecutive 1 ms pulses at 40 Hz from each experiment (PV^+^: *n* = 15 of 18; SST^+^: *n* = 19 of 22). Bold line indicates the population average. Thinner lines indicate individual experiments. Shaded area represents the SEM. Arrows indicate initial negative peak. ***I–K***, Peak frequency of oscillation before (Pre (Off)), during (On), and after (Post (Off)) light stimulation for PV^+^ with 1 ms pulse width (***I***; *n* = 18 of 18), PV^+^ with 5 ms pulse width (***J***; *n* = 13 of 13; several overlapping traces), and SST^+^ with 1 ms pulse width (***K***; *n* = 19 of 22). Experiments entrained at 20 Hz reflect suppression of alternate γ cycles.

The experiments using photoinhibition indicate that the generation of γ oscillations in hippocampal area CA3 involves the endogenous recruitment of both PV^+^ and SST^+^ interneurons. In order to test whether the activation of PV^+^ or SST^+^ interneurons is sufficient to entrain the hippocampal network at γ frequencies, we next examined cell type-specific photoexcitation using ChR2 (Nagel et al., 2003; Boyden et al., 2005). Injection of AAV-ChR2-mCherry produced similar expression patterns as Arch in both PV- and SST-Cre mouse lines (Fig. 3*A*,*B*). Photoexcitation of ChR2-expressing PV^+^ interneurons at 40 Hz (1-5 ms pulse width) reliably evoked spikes throughout pulse trains (median spike rate [interquartile range (IQR)] = 40.5 [40.3, 43.3] Hz; median spike fidelity [IQR] = 1.0 [1.0, 1.0]; *n* = 5; Fig. 3*C*,*D*), and entrained ongoing oscillations in 14 of 18 experiments (>2 mW; *n* = 12 at 5.5 mW, *n* = 6 at 2.2 mW, merged because of similar effects; Fig. 3*E*,*G*,*I*). In the remaining 4 of 18 experiments, the ongoing oscillations were not entrained (Fig. 3*I*). This effect was likely observed because of low ChR2 expression, as pulses with longer width (5 ms) entrained the oscillation in the same experiments (Fig. 3*J*). Thus, PV^+^ interneurons are sufficient to synchronize the hippocampal network at γ frequencies.

Rhythmic photoexcitation of SST^+^ interneurons evoked spikes throughout pulse trains (median spike rate [IQR] = 41.7 [40.4, 71.3] Hz; median spike fidelity [IQR] = 1.0 [1.0, 1.0]; *n* = 13; Fig. 3*C*,*D*). This stimulation pattern reliably entrained ongoing oscillations in 19 of 22 experiments (>2 mW; *n* = 13 at 5.5 mW, *n* = 9 at 2.2 mW, merged because of similar effects; Fig. 3*F*,*H*,*K*). In the remaining 3 of 22 experiments, oscillations were abolished during 40 Hz photoexcitation. These results indicate that transient activation of SST^+^ dendrite-targeting interneurons is also sufficient to synchronize the hippocampal network at γ frequencies. Activation of PV^+^ and SST^+^ interneurons produced opposite deflections in the pulse-locked waveform of the LFP recorded in the stratum pyramidale (Fig. 3*E-H*), as might be expected from the somatodendritic profile of their axon terminations. However, activation of SST^+^ interneurons was sometimes accompanied by an initial fast negative component (Fig. 3*H*), which was reminiscent of a population spike arising from the synchronized firing of excitatory cells in the hippocampus (Andersen et al., 1971; Wierenga and Wadman, 2003), despite the sparsity of SST^+^ axons in this layer.

**Figure 4.**
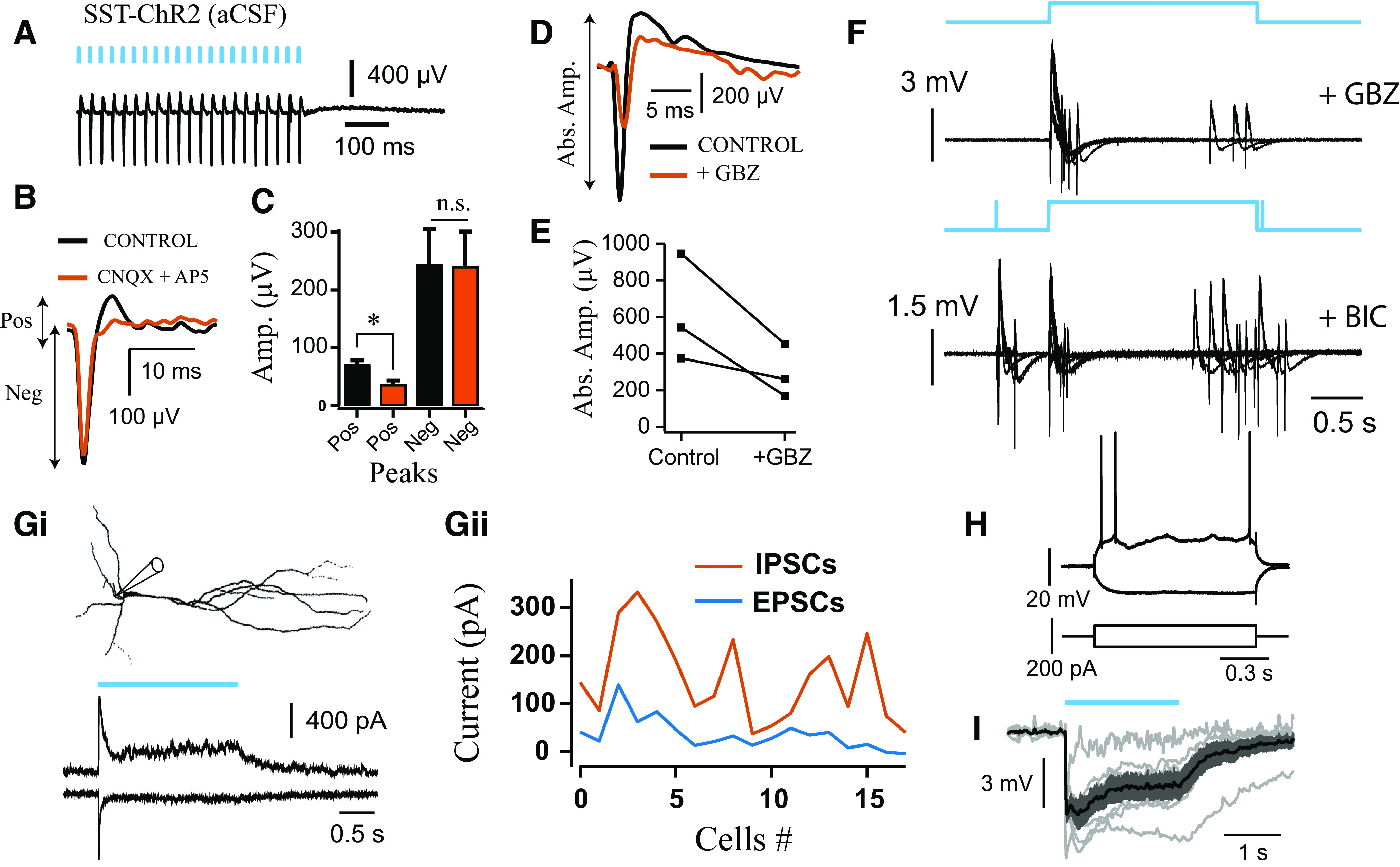
Network excitation arising from photoexcitation in SST-cre mice. ***A***, LFP responses to 40 Hz stimulation in slices from SST-ChR2 mice recorded in aCSF (1 ms pulse width; 5.5 mW). ***B***, Average waveform before (black) and after application of 20 μm CNQX and 40 μm AP5 (orange). ***C***, Effect of ionotropic glutamate receptor (iGluR) blockers on the amplitude of negative (*t* = 0.61, *p* = 0.58, n.s., not significant) and positive peaks (*t* = 3.49, *p = 0.03, *n* = 4; paired *t* test). iGluR blockers used: 20 μm CNQX, 40 μm AP5, *n* = 3; 20 μm CNQX, *n* = 1. ***D***, Average waveform before (black) and after application of 10 μm gabazine (GBZ; orange). ***E***, Effect of GBZ on absolute amplitude (*n* = 3). ***F***, Photoexcitation of SST^+^ interneurons induces epileptiform bursts during following GABA_A_R blockage (*n* = 2 at 20 μm bicuculine [BIC], *n* = 2 at 10 μm GBZ). ***Gi***, Voltage-clamp recording from putative pyramidal cell during photoactivation of SST interneurons (1.53 mW), and held at 0 mV (top) and –70 mV (bottom) to isolate IPSCs and EPSCs, respectively. ***Gii***, Across all cells, the mean EPSCs (blue) were smaller than IPSCs (orange) during SST^+^ interneuron photoactivation (*n* = 18). ***H***, ***I***, Perforated patch current-clamp recordings from putative CA3 pyramidal cell in SST-cre mice expressing ChR2-mcherry, showing responses to depolarizing and hyperpolarizing current steps (***H***), and hyperpolarization in response to light stimulation (1.53 mW; *n* = 6). Gray traces represent individual cells. Black trace represents the average and dark gray shaded area the SEM.

To study the SST^+^-induced waveform in isolation, we repeated the same experiment in quiescent slices, perfused only with aCSF. Blue light pulses (1 ms width) at 40 Hz induced strong pulse-locked field responses with fast-negative deflections, which were resistant to glutamate receptor blockers (Fig. 4*A-C*), but were followed by a glutamate receptor-mediated positive deflection. Application of GABA_A_ receptor (GABA_A_R) blockers reduced the overall amplitude of the phase-locked field responses (*n* = 3; Fig. 4*D*,*E*). Furthermore, application of GABA_A_R blockers led to light-induced epileptiform bursts (*n* = 4; Fig. 4*F*) during 2 s sustained photoexcitation. These results suggest that SST^+^ interneuron photoexcitation generates network excitation that is not mediated through GABA_A_Rs, at the onset of light illumination. We did not observe ChR2 expression in CA3 pyramidal neurons during whole-cell recordings, but they did receive weak EPSCs throughout light illumination (Fig. 4*G*). Light-evoked IPSCs were always larger than evoked EPSCs, and perforated patch-clamp recordings in current-clamp mode revealed that pyramidal cells were inhibited during SST^+^ interneuron photoexcitation (*n* = 18; Fig. 4*G–I*). Activation of GABA_B_R may contribute to the dominant effect of membrane hyperpolarization, as these receptors were blocked by QX-314 during voltage-clamp recordings. However, this does suggest that there are some off-target effects in slices from SST-ChR2 mice.

### Sustained activation of PV^+^ interneurons suppresses Cch-induced γ oscillations

**Figure 5.**
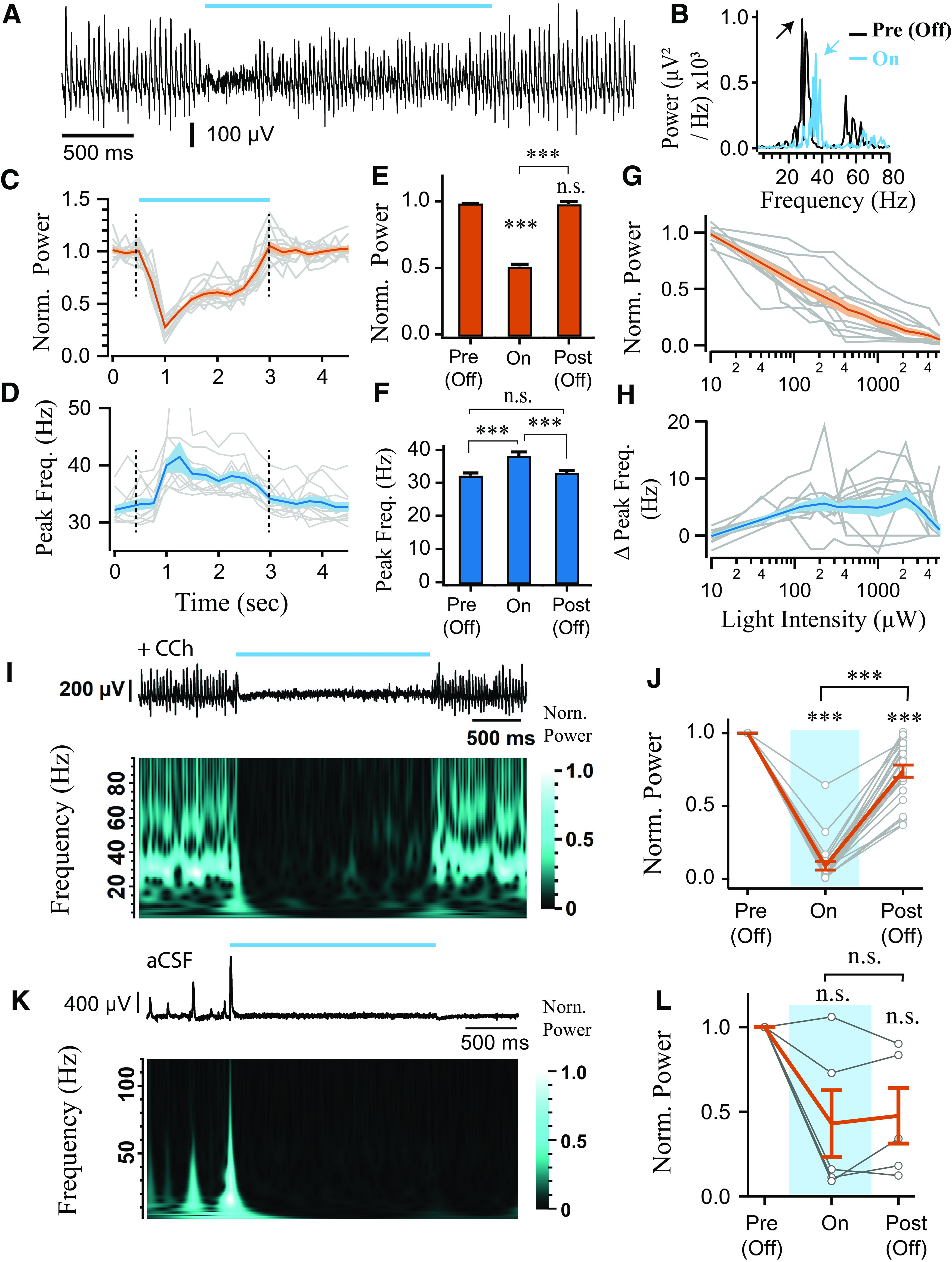
Sustained photoexcitation of PV^+^ interneurons decreases the power and increases the frequency of Cch-induced γ oscillations. ***A***, ***B***, Representative LFP recordings from CA3 area illustrating effect of PV^+^ interneurons photoexcitation (155 μW) on γ oscillations (***A***), along with its respective power spectrum (***B***; arrows indicate power spectrum peaks). ***C***, ***D***, Time course of normalized power area (***C***) and peak frequency (***D***), each calculated in 0.5 s bins across experiments (*n* = 12). ***E***, Mean changes in normalized power area. ***F***, Mean peak frequency (repeated-measures ANOVA: *F*_(1.14,12.50)_ = 44.14, *p* < 0.001). ***G***, ***H***, Normalized power area (***G***) and changes in peak frequency (***H***) plotted against light intensity (*n* = 12). ***I***, Strong and sustained blue light illumination (5.5 mW) induces a collapse of Cch-induced oscillations, as seen in LFP traces (top) and normalized wavelet spectrum (bottom). ***J***, Effect of strong illumination on mean normalized power area (*n* total = 23; *n* = 14 at 5.5 mW and *n* = 9 at 2.2 mW). ***K***, ***L***, Strong and sustained blue light illumination does not induced increases in network activity in aCSF. ***p < 0.001. n.s., not significant, *p* ≥ 0.05. Solid brackets represent paired *t* tests. Asterisks above symbols or bars represent one-sample *t* test versus normalised baseline. Gray lines indicate single experiments.

We used two patterns of sustained activation in slices from PV-ChR2 mice: light steps to drive tonic firing and fully modulated sine waves at 8 Hz to mimic excitatory input during theta-frequency oscillations (Buzsáki, 2002). In a subset of light step experiments, we recorded ongoing γ oscillations in the LFP while tonically driving PV^+^ interneurons at increasing strengths across trials (by changing the levels of blue light illumination, 10-5500 μW). The change in power between baseline and light activation period was measured at each light intensity level. We then obtained the response level at which the power changed by half of the maximum for each experiment (half-maximal response). For half-maximal response trials, photoexcitation of PV^+^ interneurons (2 s) consistently decreased the power area (0.52 ± 0.016 compared with baseline, *t* = −29.56, *p* < 0.001, one-sample *t* test; Fig. 5*A-C*,*E*) and increased the peak frequency (from 32.70 ± 0.793 Hz [baseline] to 38.76 ± 1.094 Hz, *t* = 8.21, *p* < 0.001, paired *t* test; Fig. 5*D*,*F*). Furthermore, there was a progressive decrease in power (*r* = −0.84, *n* = 121 values, *t* = 17.00, *p* < 0.001; Fig. 5*G*) and increase in frequency (*r* = 0.49, *n* = 100 of 121 values, *t* = 5.60, *p* < 0.001; Fig. 5*H*) as the light intensity increased. In order to estimate the maximal effect of PV^+^ interneuron stimulation, we pooled experiments using strong light intensity illumination (>2 mW, including cases where light intensity-response curves were not assessed; *n* = 14 at 5.5 mW, *n* = 9 at 2.2 mW). Overall, strong light illumination caused a substantial decrease in the normalized power area (0.09 ± 0.029, *t* = 31.07, *p* < 0.001, one-sample *t* test; Fig. 5*I*,*J*) and abolished the oscillations in most experiments (17 of 23). These results indicate that progressive upregulation of PV^+^ interneuron activity decreases γ power and increases the frequency until the rest of the hippocampal network is fully silenced.

**Figure 6.**
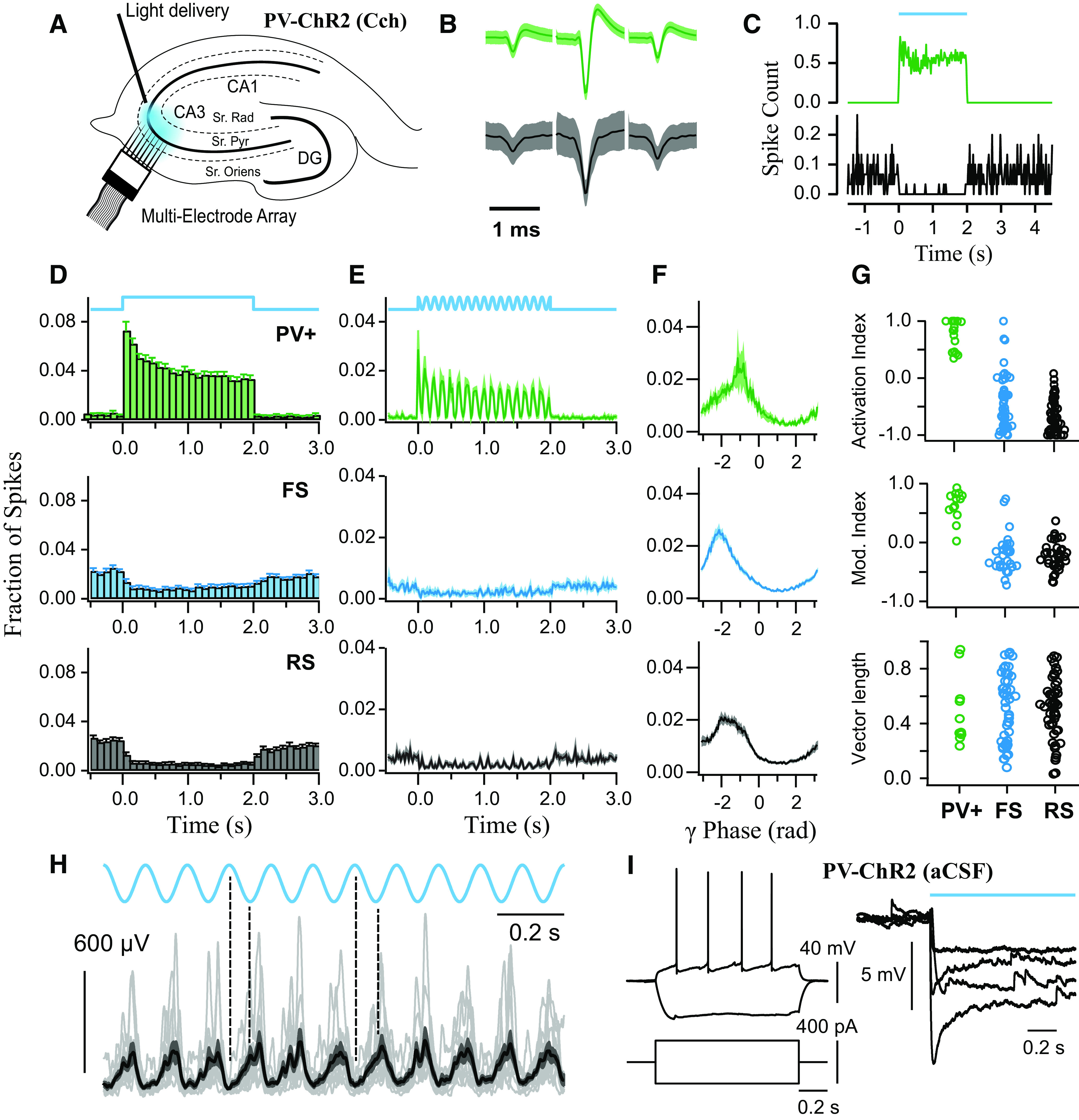
Multiunit recordings during PV^+^ interneuron sustained photoexcitation in hippocampal slices with Cch-induced γ oscillations. ***A***, Schematic diagram of the hippocampus illustrating MEA recordings during blue light illumination (5.5 mW) in CA3. ***B***, Representative average spike waveforms of PV^+^ (single-unit; green) and RS (multiunit; black) neurons. ***C***, Spike time histograms of the representative neurons during sustained light illumination. ***D***, ***E***, Mean spike time histograms during step illumination (***D***) and sinusoidal theta stimulation (8 Hz; ***E***), calculated from the fraction of total spike counts in each bin for each clustered neuron. ***F***, Spike phase histograms relative to ongoing γ-frequency oscillations, calculated from the fraction of total spike counts. ***G***, Sustained activation index (top), modulation index (middle), and vector length (bottom). ***H***, Instantaneous amplitude of the Hilbert transform during theta photoactivation (1 mW) overlaid across experiments (gray traces, *n* = 12). Black represents the mean. Dark gray represents SEM. Dotted lines indicate peaks and troughs in the light waveform. ***I***, Intracellular current-clamp recordings from pyramidal cells in aCSF, showing responses to current steps (left) and hyperpolarization in response to PV^+^ interneuron photoactivation (right; *n* = 4).

Interneurons have been shown to be particularly susceptible to depolarization block (Herman et al., 2014), and we did observe this in one of the current-clamp recordings from PV^+^ interneurons during step illumination (median spike rate [IQR] = 30.6 [8.4, 91.1] Hz; *n* = 6; excluding 1 neuron showing depolarization block). This seems unlikely to explain the effects we observe, as photostimulation in aCSF did not induce increases in network activity (Fig. 5*K*,*L*). In order to examine directly whether PV^+^ interneurons could maintain spiking with sustained photoexcitation during Cch-induced oscillations, we recorded spiking activity using a linear MEA (Fig. 6*A*). PV^+^ interneurons (spike width: 0.49 ± 0.04 ms) showed variability in spike rates (median spike rate [IQR] = 44.5 [17.3, 108.8] Hz; *n* = 18), but this activity was maintained during sustained illumination (5.5 mW; median sustained activation index [IQR] = 0.87 [0.46, 1], *Z* = 171, *p* < 0.001, *n* = 18, one-sample Wilcoxon signed rank test; analysis performed on last second of trial), and was associated with decreased activity of regular spiking (RS; −0.72 [−0.92, −0.40]; *Z* = 2, *p* < 0.001, *n* = 53, one-sample Wilcoxon signed rank test; *z* = 65.7, *p* < 0.001, compared with PV^+^ interneurons, Kruskal–Wallis test followed by *post hoc* Dunn's test with Bonferroni correction for multiple comparisons) and fast-spiking cells (FS; −0.52 [−0.82, −0.12]; *Z* = 141 *p* < 0.001, *n* = 49, one-sample Wilcoxon signed rank test; *z* = 50.1, *p* < 0.001, compared with PV^+^ interneurons, Kruskal–Wallis test followed by *post hoc* Dunn's test with Bonferroni correction for multiple comparisons) (Fig. 6*B–D*,*G*). Current-clamp recordings from putative pyramidal neurons confirmed that light stimulation produced sustained membrane hyperpolarization (Fig. 6*I*). These results are consistent with increased PV^+^ interneuron activity during light illumination that leads to reduced activity in hippocampal principal cells.

**Figure 7.**
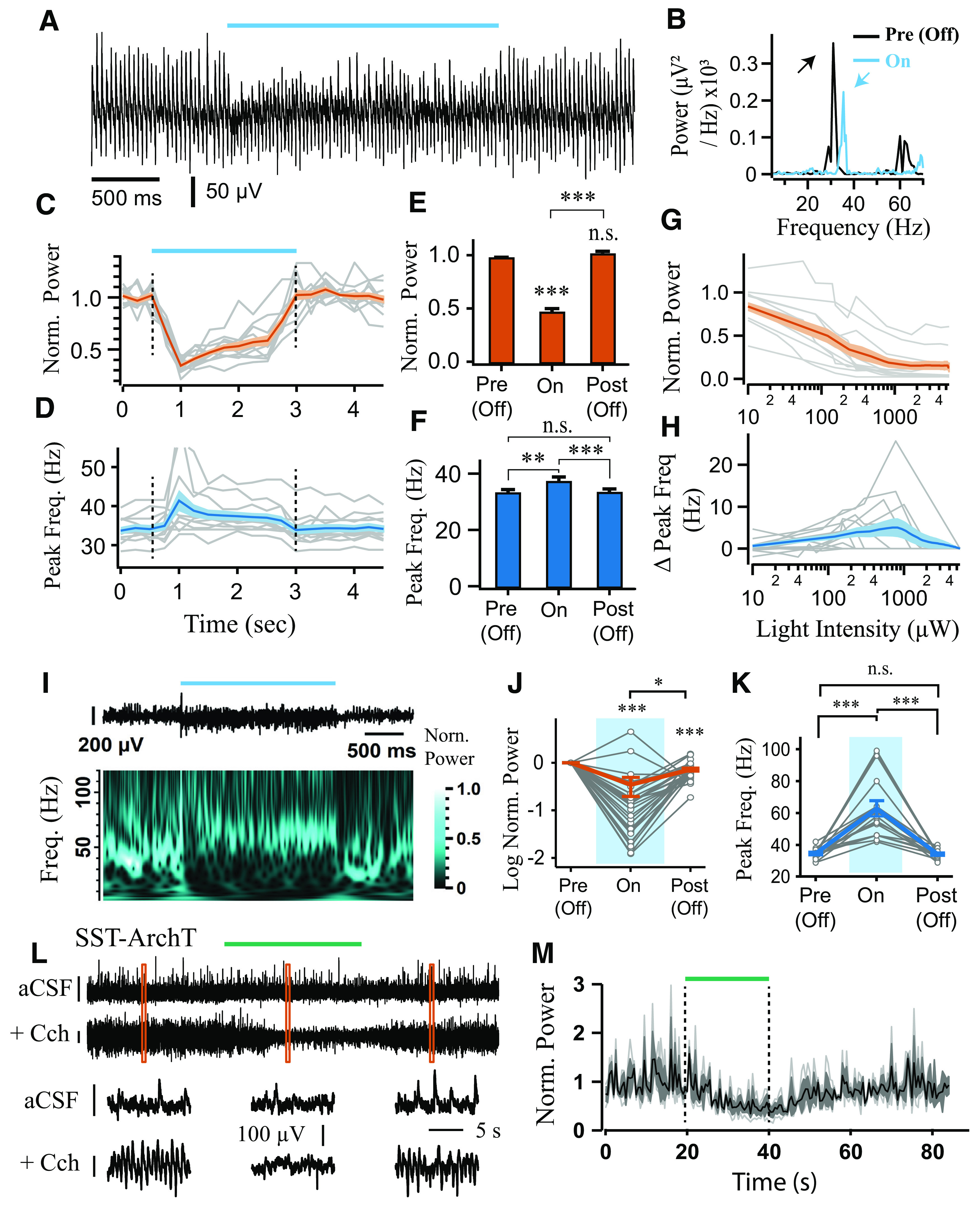
Sustained photoexcitation of SST^+^ interneurons decreases the power and increases the frequency of Cch-induced γ oscillations, but can also induce high-frequency oscillations. ***A***, ***B***, Representative LFP recordings from CA3 area illustrating effect of SST^+^ interneuron photoexcitation (155 μW) on γ oscillations (***A***), along with the respective power spectra (***B***; arrows indicate peaks in the power spectra). ***C***, ***D***, Time course of normalized power area (***C***) and peak frequency (***D***), each calculated in 0.5 s bins across experiments (*n* = 12). ***E***, Mean changes in normalized power area. ***F***, Changes in mean peak frequency (repeated-measures ANOVA: *F*_(1.05,11.59)_ = 15.05, *p* = 0.002). ***G***, ***H***, Normalized power area (***G***) and changes in peak frequency (***H***) plotted against light intensity (*n* = 12). ***I***, Strong and sustained blue light illumination (5.5 mW), which does not cause the collapse of Cch-induced oscillations, induces high-frequency oscillations, as seen in LFP traces (top) and normalized wavelet spectrum (bottom). ***J***, Normalized power during SST^+^ interneuron cell photoactivation (*n* = 31). ***K***, Peak frequency of oscillations that were not abolished during strong light illumination (*n* remaining = 16 of 31: *n* = 4 at 5.5 mW and *n* = 12 at 2.2 mW; repeated-measures ANOVA: *F*_(1.03,15.39)_ = 31.45, *p* < 0.001). Changes in peak frequency were analyzed using repeated-measures ANOVA, followed by *post hoc* paired *t* tests with correction for multiple comparisons. ***L***, Responses to laser illumination (∼18.6 mW) in SST-cre mice expressing ArchT-GFP with and without the presence of Cch. Orange squares represent the bottom sections of LFP that were magnified. ***M***, Changes in normalized power area calculated in 1 s bins across experiments (*n* = 3) in aCSF only. Dotted lines indicate the duration of laser illumination. *p < 0.05, **p < 0.01, ***p < 0.001. n.s., not significant, *p* ≥ 0.05. Solid brackets represent paired *t* tests. Asterisks above symbols or bars represent one-sample *t* test versus normalised baseline. Gray lines indicate single experiments.

During 8 Hz sinusoidal modulation of PV^+^ interneurons, the instantaneous γ magnitude, assessed using the Hilbert transform, was found to be negatively correlated with light intensity in agreement to light step experiments (across all experiments, Pearson correlation, mean *r* = −0.51 ± 0.04, *t* > 23.2, *p* < 0.001, *n* = 12) (Fig. 6*H*). During MEA recordings, spike rates of PV^+^ interneurons correlated positively with theta-frequency changes in light intensity (median rank correlation coefficient [IQR] = 0.75 [0.55, 0.83], *Z* = 120, *p* = 0.001, *n* = 15, one-sample Wilcoxon signed rank test), while negative correlations were found for the spike rates of RS (−0.19 [−0.37, −0.09]; *Z* = 100, *p* < 0.001, *n* = 43, one-sample Wilcoxon signed rank test; *z* = 48.3, *p* < 0.001, compared with PV^+^ interneurons, Kruskal–Wallis test followed by *post hoc* Dunn's test with Bonferroni correction for multiple comparisons) and FS cells (−0.19 [−0.39, −0.01]; *Z* = 232, *p* = 0.006, *n* = 42, one-sample Wilcoxon signed rank test; *z* = 45.7, *p* < 0.001, compared with PV^+^ interneurons, Kruskal–Wallis test followed by *post hoc* Dunn's test with Bonferroni correction for multiple comparisons) (Fig. 6*E*). All PV^+^ interneurons recorded during ongoing slow γ oscillations showed significant phase-coupling (*p* < 0.05, Rayleigh test), with a mean spike phase of −1.8 [−2.3, −0.9] radians (second-order mean [95% CIs]; *n* = 10; Fig. 7*F*,*G*). The PV^+^ interneurons fired at a significantly later phase of the oscillation than the RS cells (*F*_(2,55)_ = 5.36, *p* = 0.007, two-sample Hotelling test). These findings indicate that PV^+^ interneurons are synchronized within slow γ, and that a transient increase in PV^+^ interneuron activity causes a rapid and reversible decrease in the power of the Cch-γ oscillations and firing rates of other neurons.

### Sustained activation of SST^+^ interneurons induces fast γ oscillations

**Figure 8.**
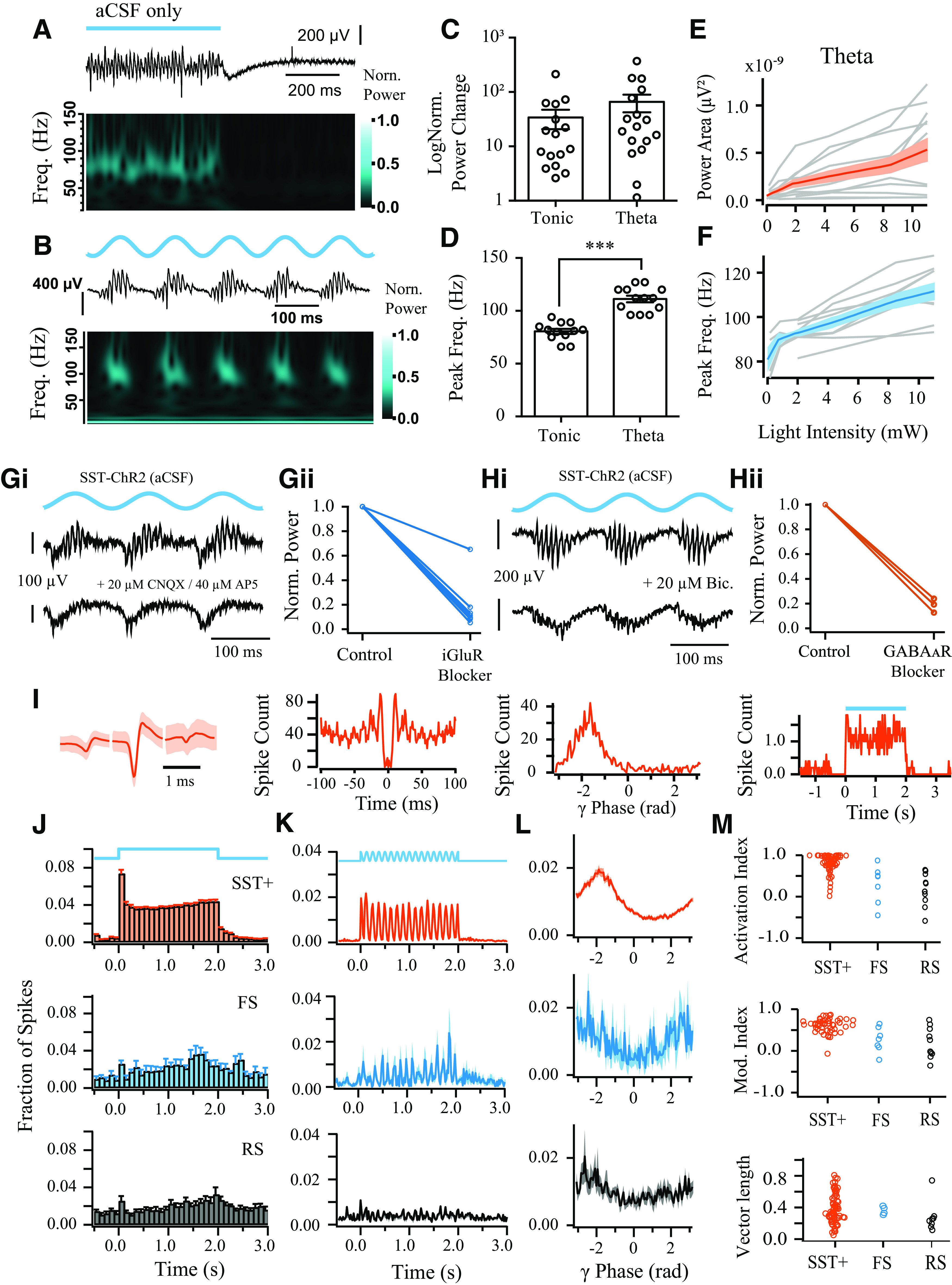
Photoactivation of SST^+^ interneurons induces *de novo* oscillations in the absence of Cch. ***A***, ***B***, Representative LFP recordings from CA3 illustrating induction of high-frequency oscillations by step (***A***) and theta-modulated (***B***) blue light illumination (10 mW), as seen in LFP traces (top) and normalized wavelet spectrum (bottom). ***C***, Change in log power compared with baseline during step (*n* = 16) and theta-modulated blue light illumination (*n* = 17). ***D***, Peak frequency of the *de novo* oscillations is higher when induced by theta compared with tonic stimulation. ***p < 0.001 (two-sample *t* test). ***E***, ***F***, Power area (***E***) and peak frequency (***F***) plotted against light intensity of theta photoactivation (*n* = 12). Black line indicates the mean response. Dark-gray shaded area represents SEM. ***G***, ***H***, Pharmacology of *de novo* oscillations induced by sinusoidal blue light illumination in SST-cre mice expressing ChR2-mcherry (1-10 mW). Representative LFP recording in CA3 before (top) and after (bottom) application of (***Gi***) iGluR blockers and (***Hi***) GABA_A_R blockers. Power area change before (control) and after application of (***Gii***) iGluR blockers and (***Hii***) GABA_A_R blockers. iGluR blockers used are as follows: 20 μm CNQX, 40 μm AP5, *n* = 3; 10 μm CNQX, 20 μm AP5, *n* = 1; 20 μm CNQX, *n* = 1; 3 mm kynurenic acid, *n* = 3. GABA_A_R blockers are as follows: 20 μm bicuculline, *n* = 2; 20 μm gabazine, *n* = 1. ***I***, Representative multiunit recordings of SST^+^ interneuron activity during step photoexcitation, showing average spike waveform, autocorrelation, phase, and step histogram. ***J***, ***K***, Mean spike time histograms during step illumination (***J***) and sinusoidal theta stimulation (8 Hz; ***K***), calculated from the fraction of total spike counts in each bin for each clustered neuron. ***L***, Spike phase histograms relative to ongoing γ-frequency oscillations, calculated from the fraction of total spike counts. ***M***, Sustained activation index (top), modulation index (middle), and vector length (bottom).

We obtained the light intensity response curves with light steps in slices from SST-ChR2 mice and observed similar results as in PV-ChR2 experiments. Sustained light illumination decreased the power (0.49 ± 0.029, *t* = 17.53, *p* < 0.001, one-sample *t* test; Fig. 7*A–C*,*E*) and increased the frequency at half-maximal response (from 34.08 ± 0.954 Hz during baseline period to 38.17 ± 1.400 Hz, *t* = 3.658, *p* < 0.01, paired *t* test; Fig. 7*D*,*F*). Moreover, as the light intensity increased, the power progressively decreased (*r* = −0.66, *n* = 107 values, *t* = 9.11, *p* < 0.001; Fig. 7*G*), and frequency progressively increased (*r* = 0.71, *n* = 56 of 107 values, *t* = 7.41, *p* < 0.001; Fig. 7*H*). It is perhaps not surprising that excitatory networks can be suppressed by photoexcitation of GABAergic interneurons. However, different responses were revealed when we assessed the effects of strong photoexcitation of SST^+^ interneurons on Cch-induced γ oscillations (light-intensity response curves were performed in a subset of experiments; *n* = 18 slices at 5.5 mW and *n* = 13 slices at 2.2 mW, merged). Similar to PV-ChR2 step experiments, the γ power was reduced during light stimulation compared with baseline period (0.34 ± 0.150, *t* = −4.39, *p* < 0.001, one-sample *t* test; Fig. 7*I*,*J*); and in approximately half of the experiments, γ oscillations were fully abolished (*n* = 16 of 31 slices). In contrast, in experiments where the oscillations persisted, their frequency increased strongly from 34.63 ± 0.836 Hz during baseline to 62.75 ± 4.921 Hz during light illumination (*n* = 15 of 31 slices; *t* = 5.61, *p* < 0.001, paired *t* test; Fig. 7*I–K*). These fast γ oscillations occurred most reliably in slices for which the light-intensity response curves were not obtained. In order to test whether SST^+^ interneuron photoexcitation alone is sufficient to induce oscillations, as opposed to simply increasing the frequency of ongoing activity, we repeated the same experiments in the absence of Cch. Sustained photoexcitation of SST^+^ interneurons induced *de novo* oscillations in the fast γ-band range with peak frequency of 80.5 ± 2.48 Hz (12 of 16 slices; Fig. 8*A*,*C*,*D*). Isolating the CA3 area from DG did not prevent the generation of *de novo* oscillations (*n* = 3 slices).

Furthermore, sinusoidal light activation at 8 Hz (theta photoexcitation) also induced robust oscillations with higher peak frequency than the tonic activation 111.2 ± 3.15 Hz (13 of 17 slices; *t* = 7.64, *p* < 0.001, two-sample *t* test; Fig. 8*B–D*). This is consistent with previous experiments showing that transient light activation induces higher frequency oscillations than sustained illumination (Butler et al., 2016; Betterton et al., 2017). Furthermore, the power (*r* = 0.67, *n* = 70 values, *t* = 7.52 *p* < 0.001) and frequency (*r* = 0.77, *n* = 48 of 70 values, *t* = 8.20, *p* < 0.001) of the *de novo* oscillations progressively increased as the light intensity of theta photoexcitation was elevated (Fig. 8*E*,*F*). This monotonic increase in peak frequency contrasts with the properties of oscillations induced by photoexcitation of principal cells in the hippocampus, where the frequency of the oscillations remains relatively constant within the slow γ band across light intensities (Butler et al., 2016, 2018; Betterton et al., 2017). Therefore, SST^+^ interneuron photoexcitation in CA3 appears to induce a distinct type of γ activity.

The fast γ oscillations that emerge during sustained photoexcitation of SST^+^ interneurons could reflect the intrinsic synchronization of SST^+^ networks, but there are a number of possible scenarios in which this stimulation paradigm could lead to the activation of other hippocampal microcircuits involving network excitation. Depolarizing GABA could contribute to recruitment of postsynaptic targets, but perforated patch recordings from hippocampal cells in stratum pyramidale (aCSF only) showed that they were hyperpolarized by light illumination (Fig. 4*H*,*I*). Alternatively, network excitation and oscillogenesis could emerge following depolarization block of SST^+^ interneurons, and subsequent disinhibition, but direct photoinhibition of SST^+^ interneurons was not able to generate *de novo* oscillations (Fig. 7*L*,*M*). However, the power of the light-induced oscillations was markedly reduced following block of either fast excitation or inhibition (Fig. 8*G*,*H*). This suggests that the light-induced oscillations recorded in LFP do not emerge solely from the GABAergic activity of SST^+^ interneurons.

Current-clamp recordings from SST^+^ interneurons during step illumination revealed that depolarization block was quite common in these cells that were located close to the surface (median spike rate [IQR] = 43.8 [33.4, 75.7] Hz; *n* = 8; excluding 7 showing depolarization block). To directly test whether photoexcitation of SST^+^ interneurons leads to a dominant effect of depolarization block during ongoing γ oscillations, and whether photoexcitation is associated with net increases or decreases in the spiking activity of other neurons in the network, we performed MEA recordings (Fig. 8*I-M*). We found that SST^+^ interneurons (spike width: 0.69 ± 0.03 ms) displayed robust activation (median spike rate [IQR] = 49.2 [29.5, 79.8] Hz; *n* = 68) that was sustained throughout the course of step stimulation (median sustained activation index [IQR] = 0.90 [0.76, 0.98], *Z* = 2346, *p* < 0.001, *n* = 68, one-sample Wilcoxon signed rank test; Fig. 8*I*,*J*,*M*), and faithfully followed the 8 Hz sine stimulation (median rank correlation coefficient [IQR] = 0.63 [0.52, 0.72], *Z* = 1484, *p* < 0.001, *n* = 54, one-sample Wilcoxon signed rank test; Fig. 8*K*,*M*). All but three of the SST^+^ interneurons recorded were significantly phase-coupled to the induced fast γ-frequency oscillations (*p* < 0.05, Rayleigh test), with a mean spike phase of −2.0 [−2.1, −1.8] radians (second-order mean [95% CIs]; *n* = 65; Fig. 8*L,M*). The RS and FS cells showed significantly weaker modulation (Fig. 8*J,K*) but did not appear to be suppressed as in the PV-ChR2 experiments, and rather showed an insignificant trends toward both increased activity during step illumination (median sustained activation index [IQR]; RS: 0.12 [−0.08, 0.54], *Z* = 50, *p* = 0.13, *n* = 11; FS: 0.49 [−0.14, 0.59], *Z* = 24, *p* = 0.09, *n* = 7; one-sample Wilcoxon signed rank tests) and positive correlations with theta-frequency changes in light intensity (median rank correlation coefficient [IQR]; RS: 0.26 [−0.07, 0.53], *Z* = 49, *p* = 0.16, *n* = 11; FS: 0.28 [0.09, 0.57] (*Z* = 25, *p* = 0.06, *n* = 7, one-sample Wilcoxon signed rank tests). The majority of RS (8 of 11) and FS cells (4 of 7) were also significantly phase-coupled to the light-induced fast γ oscillations (*p* < 0.05, Rayleigh test), but did not show a consistent mean firing phase (RS: *F*_(2,8)_ = 2.3, *p* = 0.18; FS: *F*_(2,2)_ = 4.5, *p* = 0.19; parametric second-order analysis) (Zar, 1999) (Fig. 8*L*,*M*). Overall, this suggests that the dominant change in the network during the induction of fast γ oscillations is a robust increase in the spiking of SST^+^ interneurons.

To explore whether the recruitment of SST^+^ interneurons might differ between step and theta stimulation, we analyzed the maximum spike rates in the second half of the stimulation trials (20 ms bins). The maximum spike rates during theta stimulation were significantly higher than during the step stimulation (*Z* = 148, *p* < 0.001, *n* = 54; Wilcoxon signed rank test). As theta stimulation induced faster γ oscillations than step stimulation (Fig. 8*D*), this further suggests that the frequency of fast γ oscillations depends on the overall levels of SST^+^ interneuron excitation.

### Computational model of hippocampal γ oscillations including PV^+^ and SST^+^ interneurons

**Figure 9.**
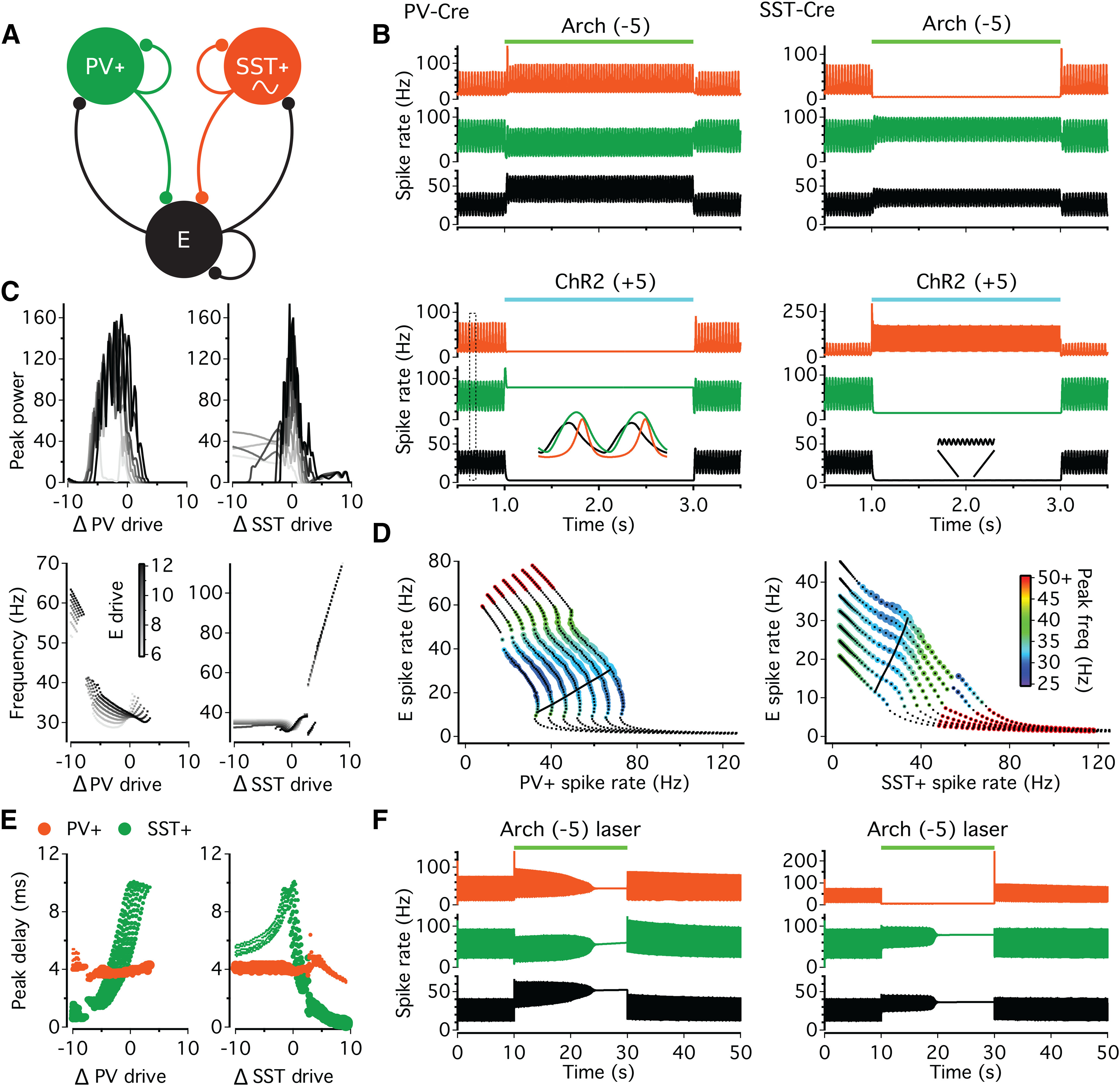
Computational model of the network effects of optogenetic modulation of interneuron activity. ***A***, Schematic of connectivity between excitatory cells (E) and PV^+^ and SST^+^ interneurons. E and PV^+^ cells were modeled using Wilson-Cowan equations and SST^+^ using equations derived from quadratic integrate-and-fire neurons, which can show intrinsic oscillations (∼). ***B***, Activity patterns observed in the three populations of cells when external drive to E cells is 10, and following inhibition (Arch; top) or excitation (ChR2; bottom) of either PV^+^ (left) or SST^+^ (right) populations. Inset left, Expansion of traces highlighted by the dashed box during the baseline period, showing temporal order of peak activity. Inset right, Expansion of E cell activity during the period shown by the dashed lines, showing small and fast oscillations. ***C***, Corresponding changes in peak power (top) and oscillation frequency (bottom), following manipulation of the external drive to PV^+^ (left) or SST^+^ (right) populations. ***D***, Plot of spike rate of E cells versus PV^+^ cells for the manipulations of PV^+^ cells (left) and plot of spike rate of E cells versus SST^+^ cells for manipulation of SST^+^ cells (right). The color of each marker represents the frequency of the network oscillation, and the size of the marker reflects peak power. Black lines join the points showing spike rates during the baseline conditions. ***E***, Delays between the peak in E cell activity and the peak in activity for PV^+^ and SST^+^ cells across different manipulations. The size of the marker reflects the corresponding spike rate. ***F***, Light-induced decreases in both excitatory drive and presynaptic release, aimed at mimicking laser stimulation of Arch, can lead to the collapse of the oscillations when applied to either the PV^+^ (left) or SST^+^ (right) populations.

In order to provide mechanistic insight into how optogenetic manipulation of PV^+^ and SST^+^ interneurons impact hippocampal network dynamics, we developed a mean firing rate model, including these two interneuronal subtypes. It has previously been shown that γ oscillations in hippocampal CA3 can be modeled using Wilson-Cowan equations in an excitatory-inhibitory feedback loop (Akam et al., 2012). We therefore used these equations to model the excitatory pyramidal cell (E) and inhibitory PV^+^ interneuron populations. The activity in the population of SST^+^ interneurons was represented using equations derived from quadratic integrate-and-fire neurons (Devalle et al., 2017), which enables oscillations within a mean firing rate model of an inhibitory network. Both interneuronal subtypes were reciprocally connected with E cells, but not with each other (Fig. 9*A*), with stronger excitatory connections to PV^+^ interneurons (Oren et al., 2006), and faster synaptic time constants in the E-PV^+^ loop (see Table 1). Moderate excitatory drive to the E population (*D_ex_*_t_ = 6-12; increments of 1) was sufficient to induce γ-frequency network oscillations (30-35 Hz; Fig. 9*B*), with little change in frequency with increasing drive (Fig. 9*C*; for ΔPV^+^ drive = 0 or ΔSST^+^ drive = 0), consistent with the effects optogenetic activation of CA3 pyramidal neurons *ex vivo* (Butler et al., 2018). As expected from the synaptic connectivity and time constants implemented in the network, peak activity occurred first in the E cells, followed by the PV^+^ cells (3.7-3.9 ms) and then SST^+^ cells (4.4-9.9 ms; decreased monotonically with increasing E drive). This is consistent with the spike delays observed for perisomatic- and dendritic-targeting interneurons recorded during Cch-induced γ-frequency oscillations *ex vivo* (Hájos et al., 2004). Oscillations were also observed at higher levels of drive to the E cells (*D_ex_*_t_ >12), but the peak in SST^+^ activity began to precede the peak in PV^+^ activity because of the voltage-dependent acceleration of SST^+^ activation. The effects of manipulating interneuronal drive were thus examined across the range of conditions that appeared to best approximate the activity in *ex vivo* slices (E cells: *D_ex_*_t_ = 6-12).

To mimic the effects of optogenetic manipulation using LEDs, a select interneuronal population received step changes in external drive (2 s), which varied from −10 to 10 across trials, with increments of 0.2. Moderate inhibition of the PV^+^ cells (*D_ex_*_t_ = −0.2 to −5.4) was ineffective at silencing PV^+^ cells and abolishing oscillations (Fig. 9*B-D*) because of corresponding increases in recurrent excitation. However, the increases in the firing rates of E and SST^+^ did have variable effects on network dynamics. Under some conditions, the increased activity in the slower E-SST^+^ loop reduced the frequency of network oscillations (Fig. 9*C*). However, the delay between E and SST^+^ activity decreased with increasing activation (Fig. 9*E*), which tended to increase oscillation frequency, and so increases or decreases in oscillation frequency were observed depending on the initial conditions and degree of PV^+^ photoinhibition (Fig. 9*C*,*D*). Progressively stronger inhibition of PV^+^ cells was capable of at first disrupting rhythmicity, and eventually allowing sufficient disinhibition of E cells to trigger fast rhythmic activity in the SST^+^ cells (Fig. 9*C*,*D*). In contrast, excitation of PV^+^ cells readily silenced E cells, and thereby abolished oscillatory activity (Fig. 9*B-D*). These manipulations of PV^+^ cells had variable effects on oscillation frequency, but there was a significant negative correlation between peak frequency and peak power for both inhibition (Spearman's ρ = −0.48, *n* = 288, *p* < 0.001) and excitation (Spearman's ρ = −0.55, *n* = 66, *p* < 0.001).

To examine the effects biochemically silencing presynaptic PV^+^ terminals, we included a light-induced reduction in the strength of PV^+^ connections to E cells and themselves, with a maximum reduction of 50%, an onset time constant of 18.4 s, and recovery time constant of 13.1 s (El-Gaby et al., 2016). When combined with presynaptic silencing, a moderate inhibitory drive to PV^+^ cells (*D_ex_*_t_ = −5) was sufficient to gradually reduce the amplitude of γ-frequency oscillations over time, and eventually abolish rhythmic activity (Fig. 9*F*).

Applying an inhibitory drive to SST^+^ cells effectively silenced this population because of weaker feedback excitation, with a predominant effect of reducing the peak power of γ-frequency oscillations (Fig. 9*B*,*C*). For low levels of inhibition of SST^+^ cells (*D_ex_*_t_ = −0.2 to −2), there were variable effects on oscillation frequency, but stronger inhibition either produced a small but consistent increase in frequency (3.0 [2.2, 3.2] Hz; E cells: *D_ex_*_t_ = 6-11) or abolished the oscillation (E cells: *D_ex_*_t_ = 11-12), depending on the initial conditions (Fig. 9*C*,*D*). An increase in frequency of persistent oscillations was expected, as the slower synaptic time constants in the loop between E and SST^+^ cells tends to lengthen the cycle period, and there was a significant negative correlation between peak frequency and peak power (Spearman's ρ = −0.28, *n* = 274, *p* < 0.001). Combining inhibitory drive with presynaptic silencing gradually abolished the oscillation (Fig. 9*F*).

Excitation of SST^+^ cells also reduced the peak power of γ-frequency oscillations (Fig. 9*C*), with corresponding changes in frequency best characterized into three phases with increasing drive: (1) the delay in the peak activity of E and SST^+^ cells decreased (Fig. 9*E*), increasing oscillation frequency; (2) the increased activity of SST^+^ cells silenced E cells, abolishing oscillatory activity; and (3) the activity in SST^+^ cells reached sufficient levels to support intrinsic fast γ-frequency oscillations (Fig. 9*C*,*D*). Under some conditions, there were intermediate phases, such as a drop in frequency because of doublet spikes in SST^+^ activity on each cycle (E cells: *D_ex_*_t_ = 11-12; SST^+^ cells: *D_ex_*_t_ = 2.8-3.4). Overall, there was a significant negative correlation between peak power and oscillation frequency (Spearman's ρ = −0.33, *n* = 326, *p* < 0.001). This computational model of hippocampal oscillations indicates that SST^+^ interneuron sustained excitation is sufficient to generate γ oscillations under certain conditions.

## Discussion

γ oscillations depend on synchronized synaptic inhibition, and there is a wealth of evidence suggesting that perisomatic-targeting PV^+^ interneurons are critical for both current and rhythm generation (Penttonen et al., 1998; Mann et al., 2005b; Bartos et al., 2007; Oren et al., 2010; Tukker et al., 2013; Cardin, 2016; Sohal, 2016). Here, we used optogenetic manipulation of PV^+^ and SST^+^ interneurons to explore whether PV^+^ interneurons have a selective role in γ rhythmogenesis in the hippocampal CA3 *ex vivo*. Our findings suggest that disrupting interneuronal activity, via either photoinhibition or photoexcitation, generally leads to a decrease in the power and increase in the frequency of ongoing cholinergically induced slow γ oscillations. This suggests that both PV^+^ and SST^+^ interneurons play key roles in maintaining slow γ oscillations, and the key differences were that (1) γ oscillations were more readily disrupted by photoinhibition of SST^+^ rather than PV^+^ interneurons, (2) manipulation of SST^+^ interneurons modulated γ frequency more robustly than that of PV^+^ interneurons, and (3) photoexcitation of SST^+^ interneurons could also induce *de novo* fast γ oscillations. These key differences were replicated in a mean firing rate model, in which excitatory neurons and PV^+^ interneurons were connected in a strong and fast feedback loop, and modeled using Wilson-Cowan equations, and SST^+^ neurons were modeled using equations derived from quadratic integrate-and-fire neurons that support interneuronal network oscillations.

Slow γ oscillations in the hippocampal CA3 appear to be generated by synaptic feedback loops between excitatory pyramidal neurons and perisomatic-targeting interneurons, both in brain slices (Fisahn et al., 1998; Hájos et al., 2004; Mann et al., 2005b; Oren et al., 2006; Butler et al., 2018) and *in vivo* (Bragin et al., 1995; Csicsvari et al., 2003; Fuchs et al., 2007). In such feedback loops, the period of the oscillation largely reflects the effective time course of inhibitory postsynaptic potentials in the pyramidal cells, which should become shorter with smaller compound inhibitory synaptic currents and/or increased pyramidal cell excitability. The amplitude of the oscillation recorded in the LFP also reflects the amplitude of phasic inhibitory currents in pyramidal neurons (Mann et al., 2005b; Oren et al., 2010); and during spontaneous γ oscillations, there is a strong correlation between the instantaneous period and amplitude of each γ cycle (Atallah and Scanziani, 2009). One might thus expect disinhibition to decrease the amplitude and increase the frequency of γ oscillations, which is largely what we observed with photoinhibition of either PV^+^ or SST^+^ interneurons.

While photoinhibition of SST^+^ interneurons was able to reliably disrupt γ oscillations, it was necessary to use high-powered laser illumination of PV^+^ interneurons to consistently reduce γ power, and the oscillations were not abolished under our stimulation paradigms. This is not inconsistent with PV^+^ interneurons playing a key role in the synaptic feedback loops generating γ oscillations in the hippocampal CA3, as such a microcircuit should resist disinhibition. Indeed, it appears that strong laser illumination was necessary to biochemically silence PV^+^ interneuron terminals (El-Gaby et al., 2016), and thus break this feedback loop.

Our computational model supports these conclusions and provides insight into a potential mechanism for the network effects of photoinhibition. In this model, the E and PV^+^ cells are connected in a strong and fast feedback loop, which can oscillate alone at 60-70 Hz with external excitatory drive to the E cells (data not shown). The connections from E cells to SST^+^ cells are weaker and slower, and recurrent inhibition also has a slower time constant. Under baseline conditions, this loop slows the frequency of network oscillations to 30-35 Hz. However, as the SST^+^ population is modeled by macroscopic equations for quadratic integrate-and-fire neurons, membrane depolarization leads to sharp increases in firing rate, and can lead to the generation of fast γ within the SST^+^ population. Photoinhibition of SST^+^ cells reduces the influence of the slow E-SST^+^ loop, while increasing the firing rates of E and PV^+^ cells, leading to faster and weaker oscillations. Photoinhibition of PV^+^ cell firing rates is resisted by strong feedback excitation but leads to increased activity of E cells and greater synaptic excitation of SST^+^ cells. Depending on the initial conditions, this leads to increases or decreases in the oscillation frequency, which may explain the variable effects we observed *ex vivo*. It was possible to break the feedback loop by providing strong inhibitory drive to the PV^+^ cell population, which could unleash fast γ in the SST^+^ cell population. We did not observe such effects *ex vivo*, which could reflect the power of the optogenetic inhibitors and/or light penetration. However, combining more moderate inhibition with presynaptic silencing was also effective in the model.

In *ex vivo* brain slices, we also found that photoexcitation of PV^+^ or SST^+^ interneurons led to an increase in the frequency and decrease in power of γ oscillations. This might be somewhat more surprising, but our model provides a relatively simple explanation. Photoexcitation of PV^+^ cells reduces E cell activity and the recruitment of SST^+^ cells, whereas photoexcitation of SST^+^ cells enables their accelerated recruitment because of membrane depolarization. Both of these effects reduce the slowing influence of E-SST^+^ loop observed during baseline conditions.

It was recently suggested that SST^+^ interneurons, but not PV^+^ interneurons, contribute to the generation of slow γ oscillations in V1 (Chen et al., 2017; Veit et al., 2017; Hakim et al., 2018). Our results do not support an exclusive role for SST^+^ interneurons in slow hippocampal γ oscillations but are consistent with an important role for SST^+^ interneurons in γ rhythmogenesis across cortical circuits. However, SST^+^ interneurons largely target the dendritic domains of pyramidal cells; thus, it remains difficult to see how they could directly contribute to the precise timing of pyramidal cell spiking during fast brain oscillations. SST^+^ bistratified interneurons have similar properties to fast spiking PV^+^ interneurons, and also form a portion of synapses close to the soma (Somogyi and Klausberger, 2005; Muller and Remy, 2014), but have been reported to exhibit decreased GABA release under cholinergic stimulation (Gulyás et al., 2010). In our model, the dendritic location of SST^+^ is only represented by the slower time constant of inhibition, but it highlights a potentially important role of hippocampal SST^+^ interneurons in modulating the frequency of slow γ oscillations that can be expressed in E-PV^+^ circuits.

While optogenetic manipulation of SST^+^ interneurons consistently disrupted slow γ oscillations, we found that photoexcitation of SST^+^ interneurons could also induce *de novo* fast γ oscillations. These GABAergic interneurons should provide a powerful source of circuit inhibition (Somogyi and Klausberger, 2005; Taniguchi et al., 2011; Leão et al., 2012; Lovett-Barron et al., 2012, 2014; Royer et al., 2012; Pfeffer et al., 2013; Urban-Ciecko and Barth, 2016), but we found that sustained photoexcitation of SST^+^ interneurons did not significantly inhibit the activity of ChR2^–^ neurons, and that pulsed stimulation could drive network excitation. In our model, we could induce fast γ via photoexcitation of SST^+^ cells, but this was accompanied by a strong suppression of activity in E and PV^+^ cells, and so it is likely that there are nonspecific effects of ChR2 stimulation. There have been reports of off-target expression in juvenile SST-Cre mice (Taniguchi et al., 2011), so there is still a possibility for pyramidal cell expression that we could not detect, or even SST^+^ cells that corelease glutamate (Cattaneo et al., 2019). An alternative possibility is that robust activation of a dense plexus of SST^+^ axons in the dendritic layers is sufficient to induce spiking in pyramidal neurons via ephaptic coupling (Anastassiou et al., 2011; Ferenczi et al., 2016) or changes in extracellular ion concentration, which would be enhanced under interface recording conditions (Octeau et al., 2019), and counteract the effects of synaptic inhibition. The generation of fast γ oscillations appeared to depend on the maintenance of network excitability, as the oscillations were attenuated by block of iGluRs. However, the spiking of the majority of SST-RS neurons was only weakly coupled to the phase of light-induced fast γ oscillations, and without a consistent population spike phase preference, while light-sensitive putative SST^+^ interneurons showed reliable phase-locking. This could be consistent with fast γ oscillations representing rhythmic dendritic inhibition from SST^+^ interneurons, with only weak effects on the spike rate and timing of other neurons in the network.

The mechanism by which a network of SST^+^ interneurons might generate fast γ oscillations remains obscure. In neocortex, SST^+^ interneurons avoid inhibiting each other (Pfeffer et al., 2013), although there is evidence for sparse synaptic interactions between SST^+^ interneurons in the hippocampus (Savanthrapadian et al., 2014), and for more generic coupling via gap junctions (Baude et al., 2007). More experiments are required to resolve the mechanisms by which optogenetic manipulation of interneurons influences hippocampal γ oscillations, and whether SST^+^ neurons contribute to fast hippocampal γ oscillations during theta and non-theta states *in vivo* (Sullivan et al., 2011). However, our findings suggest that SST^+^ interneurons exert powerful control over the power and frequency of slow hippocampal γ oscillations, and contribute to the generation of fast γ states.
